# Activation of Cph1 causes ß(1,3)-glucan unmasking in *Candida albicans* and attenuates virulence in mice in a neutrophil-dependent manner

**DOI:** 10.1371/journal.ppat.1009839

**Published:** 2021-08-25

**Authors:** Andrew S. Wagner, Trevor J. Hancock, Stephen W. Lumsdaine, Sarah J. Kauffman, Mikayla M. Mangrum, Elise K. Phillips, Timothy E. Sparer, Todd B. Reynolds

**Affiliations:** Department of Microbiology, University of Tennessee, Knoxville, Tennessee, United States of America; Dartmouth College, Geisel School of Medicine, UNITED STATES

## Abstract

Masking the immunogenic cell wall epitope ß(1,3)-glucan under an outer layer of mannosylated glycoproteins is an important virulence factor deployed by *Candida albicans* during infection. Consequently, increased ß(1,3)-glucan exposure (unmasking) reveals *C*. *albicans* to the host’s immune system and attenuates its virulence. We have previously shown that activation of the Cek1 MAPK pathway via expression of a hyperactive allele of an upstream kinase (*STE11*^*ΔN467*^) induced unmasking. It also increased survival of mice in a murine disseminated candidiasis model and attenuated kidney fungal burden by ≥33 fold. In this communication, we utilized cyclophosphamide-induced immunosuppression to test if the clearance of the unmasked *STE11*^*ΔN467*^ mutant was dependent on the host immune system. Suppression of the immune response by cyclophosphamide reduced the attenuation in fungal burden caused by the *STE11*^*ΔN467*^ allele. Moreover, specific depletion of neutrophils via 1A8 antibody treatment also reduced *STE11*^*ΔN467*^-dependent fungal burden attenuation, but to a lesser extent than cyclophosphamide, demonstrating an important role for neutrophils in mediating fungal clearance of unmasked *STE11*^*ΔN467*^ cells. In an effort to understand the mechanism by which Ste11^ΔN467^ causes unmasking, transcriptomics were used to reveal that several components in the Cek1 MAPK pathway were upregulated, including the transcription factor *CPH1* and the cell wall sensor *DFI1*. In this report we show that a *cph1ΔΔ* mutation restored ß(1,3)-glucan exposure to wild-type levels in the *STE11*^*ΔN467*^ strain, confirming that Cph1 is the transcription factor mediating Ste11^ΔN467^-induced unmasking. Furthermore, Cph1 is shown to induce a positive feedback loop that increases Cek1 activation. In addition, full unmasking by *STE11*^*ΔN467*^ is dependent on the upstream cell wall sensor *DFI1*. However, while deletion of *DFI1* significantly reduced Ste11^ΔN467^-induced unmasking, it did not impact activation of the downstream kinase Cek1. Thus, it appears that once stimulated by Ste11^ΔN467^, Dfi1 activates a parallel signaling pathway that is involved in Ste11^ΔN467^-induced unmasking.

## Author summary

*Candida albicans* is a significant cause of fungal bloodstream infections. Yet, in order for it to effectively induce disease, host immune evasion must be achieved. *C*. *albicans* often accomplishes this by masking structures within its cell wall that are recognized by the host immune system, such as ß(1,3)-glucan. Thus, inappropriately exposing (unmasking) this epitope may be a way to increase immune system recognition of invading fungal cells to enhance host clearance. We have previously shown that we can induce inappropriate unmasking in *C*. *albicans* by regulated expression of a hyperactive mutant of the MAP3K *STE11* of the Cek1 MAPK pathway, and that this reduces fungal colonization during systemic infection in mice. We hypothesized that the observed virulence attenuation was a consequence of enhanced host immune system recognition caused by unmasking induced by the canonical Cek1 MAPK pathway. In this report, we found that Ste11^ΔN467^-induced unmasking was indeed mediated by one of its known downstream transcription factors, Cph1, and that its virulence attenuation *in vivo* was largely dependent on a functional immune response. Thus, leveraging the Cek1 pathway may serve as a model to study how unmasking occurs in *C*. *albicans*, and how the host responds to this phenotype *in vivo*.

## Introduction

Pathogenic fungi are a significant burden to public health, accounting for up to 1.5 million deaths annually worldwide [1]. Within all infected individuals, the health status of the host and the ability of the pathogen to successfully evade host immune system detection are closely related to the severity of the resulting infection. In a healthy host, the immune system is equipped with numerous pattern recognition receptors (PRRs) that are capable of recognizing and mounting an immune response to pathogen associated molecular patterns (PAMPs) located on the fungi [2]. The cell wall is a major source of fungal associated PAMPs, and therefore acts a vital junction in this host immune system-pathogen interaction [3,4]. Consequently, fungal pathogens have evolved regulatory systems to dictate the level of exposure of these immunogenic epitopes located within their cell wall as a means to mediate host immune system evasion.

Fungal pathogens deploy various mechanisms to successfully evade their host. Several pathogens utilize thick outer proteinaceous or carbohydrate layers to cover immunogenic epitopes within their cell walls. For example, wild-type *Candida albicans* covers (masks) immunogenic ß(1,3)-glucan, located within the central layer of its cell wall, with a dense outer layer of mannosylated proteins [5,6]. This masking facilitates immune system evasion by shielding immunogenic ß(1,3)-glucan epitopes from host PRRs, such as dectin-1, compliment receptor 3, and EphA2 located on macrophages [7–11], neutrophils [10,12,13] and host epithelia [14]. *Aspergillus fumigatus* covers immunogenic epitopes with hydrophobins or galactosaminogalactan on its conidia and hyphae, respectively, to similarly impair dectin-1 binding [15,16]. Alternatively, pathogens may also utilize extracellular enzymes that are capable of reducing the levels of exposed antigens in their cell wall. This can be observed in both *C*. *albicans* and *Histoplasma capsulatum*, which utilize the endo-1,3-ß-glucanase Eng1 to degrade exposed ß(1,3)-glucan [17,18]. *C*. *albicans* can additionally deploy the exo-1,3-ß-glucanase Xog1 to induce masking in response to lactate exposure [19].

Due to the vital interface that the cell wall plays in regulating successful immune system evasion by pathogenic fungi, disruption of antigen masking is beginning to gain attention as an alternative therapeutic approach to treating disease progression [19–21]. In this strategy, the host immune system can be leveraged to facilitate enhanced fungal clearance. With respect to Candidiasis, the applicability of this approach will benefit from an understanding of how *C*. *albicans* actively regulates its levels of exposed immunogenic epitopes, such as ß(1,3)-glucan and chitin, and how the host immune system subsequently responds to infection by the unmasked fungus. Such information could be used to attenuate the ability of *C*. *albicans* to evade host immune system detection and further enhance clearance. Recent findings are beginning to highlight the applicability of regulated activation of signal cascades to address the aforementioned questions. *C*. *albicans* has been shown to regulate its levels of ß(1,3)-glucan exposure in response to various environmental stimuli, including acidic pH, hypoxia and lactate exposure [22–24]. This change in cell wall architecture is facilitated through appropriate signal transduction cascades that are induced in response to the environmental stimulus. For example, in response to lactate exposure, *C*. *albicans* induces masking via a non-canonical signal cascade between the G-coupled-protein-receptor Gpr1 and the transcription factor Crz1 [24]. This cascade in turn influences the expression of the exo-1,3-ß-glucanase Xog1 to regulate masking [19]. Thus, regulated activation of signal cascades that mediate masking or unmasking can serve as a model to study how *C*. *albicans* regulates its homeostatic levels of exposed ß(1,3)-glucan. Furthermore, these pathways may also serve as inducible systems to study how the host immune system responds to infection with unmasked *C*. *albicans* cells *in vivo*. Similarly, our lab has shown that hyperactivation of the Cek1 Mitogen Activated Protein Kinase (MAPK) pathway, consisting of a Ste11-Hst7-Cek1 signal cascade, is capable of activating ß(1,3)-glucan exposure [25,26]. We have shown that regulated hyperactivation of the pathway via expression of a hyperactive allele of the upstream MAP3 kinase (*STE11*^*ΔN467*^) under the control of a tetracycline (*tet*)-repressible promoter causes increased ß(1,3)-glucan unmasking that elicits activation of macrophages (cytokine secretion) *in vitro* [25]. Furthermore, we have shown that activation of this pathway can be regulated *in vivo* and that mice infected with the *STE11*^*ΔN467*^ strain show prolonged survival and a ≥33-fold reduction in kidney fungal burden compared to the wild-type during systemic infection.

In this report, we utilize the controlled activation of the Cek1 MAPK pathway to assess the impact that the host immune system has on clearance of an unmasked *STE11*^*ΔN467*^ mutant during systemic infection, and further elucidate how *STE11*^*ΔN467*^ expression induces ß(1,3)-glucan unmasking in *C*. *albicans*. We found that clearance of unmasked *STE11*^*ΔN467*^ cells is largely host immune system dependent, and relies on multiple immune cells including neutrophils for proper clearance. We also present data showing that *STE11*^*ΔN467*^-induced unmasking of both ß(1,3)-glucan and chitin is mediated through the transcription factor Cph1 and that activated Cph1 induces overexpression of the cell wall sensor *DFI1*, which stimulates an unidentified parallel signaling pathway that further influences ß(1,3)-glucan exposure.

## Results

### Systemic infection with a hyperactive *STE11*^*ΔN467*^ mutant decreases organ colonization and circulatory cytokine levels in mice

We have previously shown that overexpression of a single allele of a hyperactive *STE11* mutant gene (*STE11*^*ΔN467*^) induces increased ß(1,3)-glucan unmasking and attenuates virulence in a mouse model of systemic candidiasis [25,26]. However, although we have shown that hyperactive *STE11*^*ΔN467*^ prolongs survival and significantly reduces kidney fungal burden in mice during systemic infection, its impact on colonization in other host organs is unknown. Thus, to develop a more holistic understanding of the impact that hyperactive *STE11*^*ΔN467*^ expression has on systemic disease progression, we intravenously infected mice with either wild-type *C*. *albicans* or a strain carrying a normal *STE11* allele on one chromosome and a hyperactive *STE11*^*ΔN467*^ allele under the control of a tetracycline (*tet*)-repressible promoter on the other (*STE11/P*_*tet-off*_*-STE11*^*ΔN467*^) [25,27]. The use of the *tet*-repressible promoter allows for controlled regulation of the hyperactive *STE11*^*ΔN467*^ allele. The addition of doxycycline causes the strain to act similarly to the wild-type because the *STE11*^*ΔN467*^ allele is repressed, but the remaining wild-type copy of *STE11* is still expressed. In contrast, in the absence of doxycycline the *P*_*tet-off*_ promoter overexpresses the hyperactive *STE11*^*ΔN467*^ allele and induces unmasking. Thus, if we infect mice with the *STE11/P*_*tet-off*_*-STE11*^*ΔN467*^ strain without giving the mice doxycycline in their drinking water, then the strain will be unmasked. To ensure that unmasking occurred only during infection, overnight cultures of the wild-type and *STE11/P*_*tet-off*_*-STE11*^*ΔN467*^ were grown in YPD with doxycycline to repress premature hyperactivation of the Cek1 MAPK pathway [25]. Mice were then intravenously infected with 1x10^6^ CFU/mouse of either the wild-type or *STE11/P*_*tet-off*_*-STE11*^*ΔN467*^ strain. Doxycycline was either present or absent in the drinking water of mice to regulate *P*_*tet-off*_*-STE11*^*ΔN467*^ expression. Mice were then sacrificed at 4 days post infection (d.p.i) to assess fungal burden of the kidney, liver, spleen and brain.

**Fig 1:**
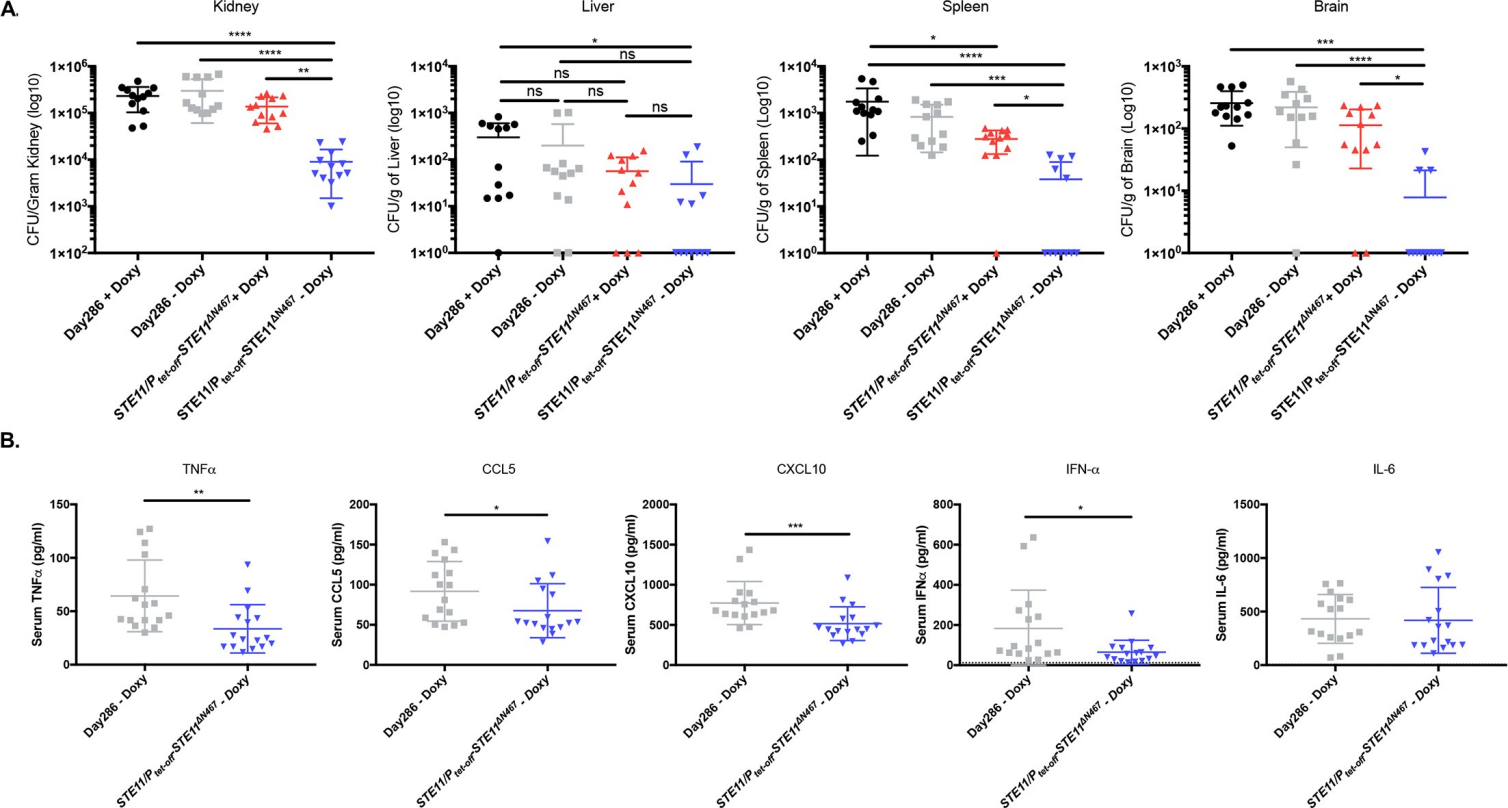
Systemic infection with a hyperactive *STE11*^*ΔN467*^ mutant attenuates fungal burden in multiple niches within a mouse host. (A) ICR mice were intravenously infected with 1x10^6^ cells of *C*. *albicans* wild-type (Day286) or the *STE11/P*_*tet-off*_*-STE11*^*ΔN467*^ strain and their kidneys, livers, spleens, and brains were harvested 4 days post infection (d.p.i.) to assess fungal burden. (n = 6 mice)(*p<0.05, **p<0.01, ***p<0.0005, ****p<0.0001, by Kruskal-Wallis test with Dunn’s multiple comparisons post-hoc analysis). (B) Serum collected 4 days post infection was assessed to determine the protein concentrations of TNFα, CCL5, CXCL10, IFNα and IL-6 via flow cytometry using the LEGENDplex cytokine bead-based array kit. (n = 8 mice)(*p<0.05, **p<0.005, ***p<0.0005, by Mann-Whitney test).

In accordance with our previous observation [25], an ~15-33-fold decrease in kidney fungal burden was observed in mice infected with *C*. *albicans* that overexpressed *STE11*^*ΔN467*^ (*STE11/P*_*tet-off*_*-STE11*^*ΔN467*^ strain without doxycycline) compared to all other conditions (Fig 1A). Furthermore, this difference was also apparent in fungal burden trends in both the spleen and brain, suggesting that hyperactive *STE11*^*ΔN467*^ expression during systemic infection impacts fungal burden in multiple host niches to which *C*. *albicans* disseminates. However, liver fungal burden trends only showed a significant difference between mice infected with *STE11/P*_*tet-off*_*-STE11*^*ΔN467*^ without doxycycline and mice infected with wild-type plus doxycycline.

In accordance with the decreased colonization levels observed in the kidneys, brains and spleens of mice infected with the *STE11/P*_*tet-off*_*-STE11*^*ΔN467*^ strain, there was also a modest reduction in several proinflammatory cytokines within the serum of mice infected with the hyperactive *STE11*^*ΔN467*^ mutant at 4 days post infection. Namely, TNFα (p<0.005), CCL5 (p<0.05), CXCL10 (p<0.0005) and IFNα (p<0.05) were found at lower levels within the serum of mice infected with the *STE11/P*_*tet-off*_*-STE11*^*ΔN467*^ strain when compared to the wild-type control (Fig 1B). However, no difference was observed in the serum concentrations of IL-6, CCL2, IL-1ß, IL-12, GM-CSF, CXCL1, IL-10, IFN-ß or IFN-γ (Figs 1B and S1). Together, this suggests that systemic infection with a hyperactive *STE11*^*ΔN467*^ mutant only modestly impacts serum concentrations of select proinflammatory cytokines at 4 days post infection.

### Virulence attenuation by hyperactive Ste11^*ΔN467*^ is host immune system dependent

**Fig 2:**
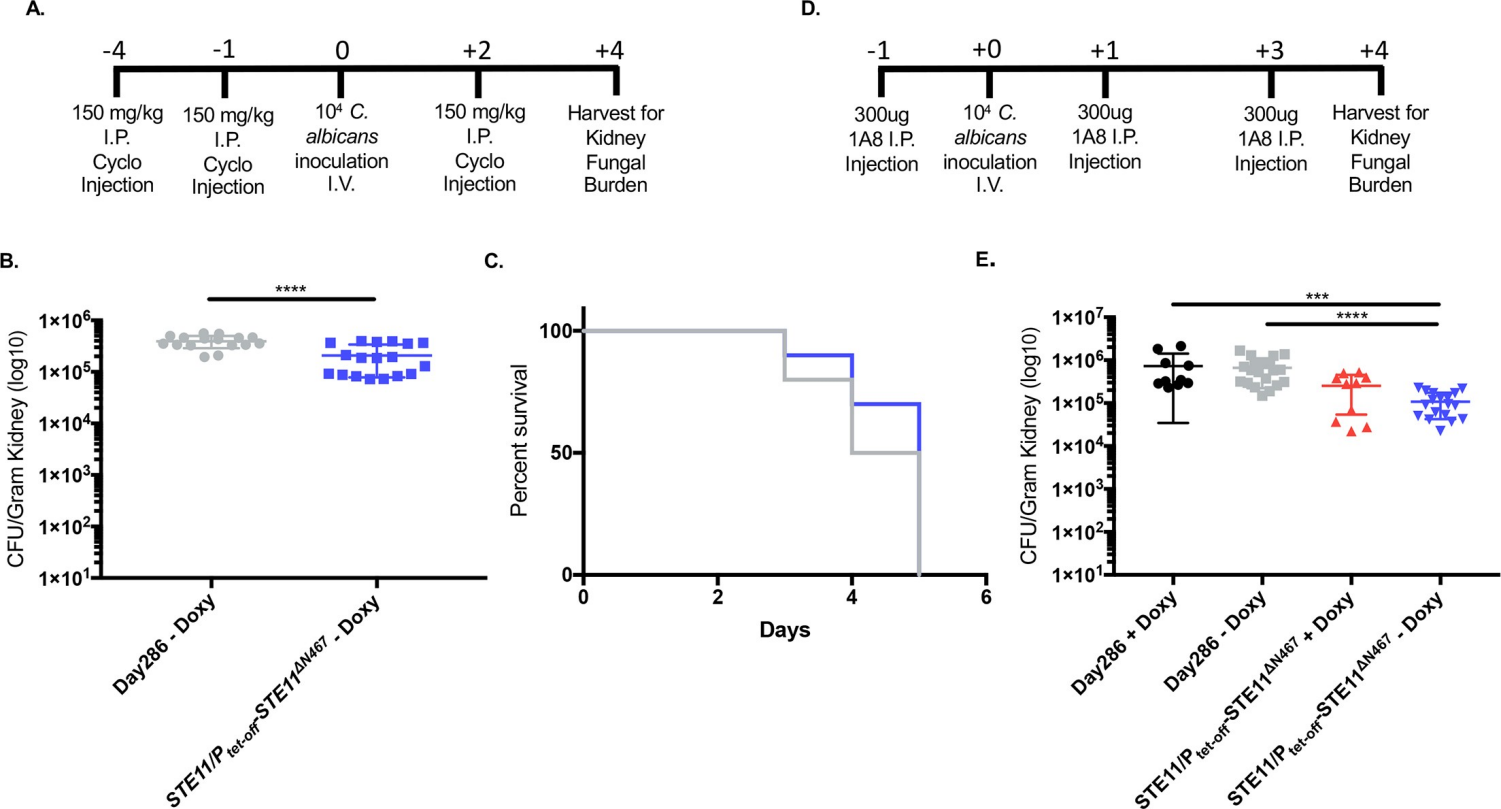
Virulence attenuation during systemic infection by the hyperactive *STE11*^*ΔN467*^ mutant is host immune system dependent. (A) ICR mice were immunosuppressed with recurring intraperitoneal injections of 150mg/kg of cyclophosphamide (Cyclo) every 3 days starting 4 days prior to infection. At day 0, mice were intravenously injected with 1x10^4^ cells of *C*. *albicans* wild-type (Day286) or the *STE11/P*_*tet-off*_*-STE11*^*ΔN467*^ strain that were previously grown in YPD containing doxycycline to repress *STE11*^*ΔN467*^ expression prior to infection. Kidneys were harvested 4 days post infection (d.p.i.) to assess fungal burden. (B) Kidney fungal burden in mice immunosuppressed with cyclophosphamide 4 d.p.i. (n = 8–9 mice) (*p<0.0001, by student’s t-test). (C) Survival curve for cyclophosphamide-immunosuppressed mice that were intravenously injected with 1x10^4^ cells of wild-type (Day286) or the *STE11/P*_*tet-off*_*-STE11*^*ΔN467*^ strain. Immunosuppression was maintained via recurring intraperitoneal injections every 3 days with 150mg/kg of cyclophosphamide starting 4 days prior to infection (day -4). (n = 10 mice) (D) Neutrophil depletion was achieved with recurring intraperitoneal injections of 300ug of anti-mouse Ly6G (1A8) antibody every 2 days starting one day prior to infection. At day 0, ICR mice were intravenously injected with 1x10^4^ cells of *C*. *albicans* wild-type (Day286) or the *STE11/P*_*tet-offf*_*-STE11*^*ΔN467*^ strain and kidney fungal burden was assessed 4 d.p.i. (E) Kidney fungal burden in neutrophil depleted mice 4 d.p.i. (+/- Doxy = addition or absence of doxycycline) (n = 5 mice for Day286 and *STE11/P*_*tet-off*_*-STE11*^*ΔN467*^ + doxycycline controls, n = 9 mice for *STE11/P*_*tet-off*_*-STE11*^*ΔN467*^ –doxycycline, and n = 10 mice for Day286 –doxycycline) (***p<0.0005 and ****p<0.0001, by Kruskal-Wallis test with Dunn’s multiple comparisons post-hoc analysis).

Although the data in Fig 1A showed a clear impact of hyperactive *STE11*^*ΔN467*^ expression on fungal burden in multiple organs during systemic infection, the mechanism driving virulence attenuation in mice was unclear. We hypothesized that the observed virulence decrease was driven by enhanced recognition and clearance of *Candida* by the immune system as a consequence of hyperactive Cek1-mediated unmasking. However, decreased growth in the host could be responsible for the phenotype as well. Therefore, to assess whether virulence attenuation by *STE11*^*ΔN467*^ is dependent on the host’s immune system, we immunosuppressed mice with cyclophosphamide prior to infection (Figs 2A and S2). This should eliminate any virulence differences that are dependent on the host’s immune response. We then inoculated cyclophosphamide-treated mice with 1x10^4^ CFU/mouse of either the wild-type strain as a control or the *STE11/P*_*tet-off*_*-STE11*^*ΔN467*^ strain. A lower infection dose was used to account for the increased susceptibility of immunosuppressed mice. Mice were not given doxycycline in their drinking water to permit hyperactive *STE11*^*ΔN467*^ expression and all mice were sacrificed at 4 days post infection (d.p.i) to measure kidney fungal burden.

Mice infected with wild-type *C*. *albicans* exhibited an average fungal burden of 3.94x10^5^ CFU g^-1^ kidney, while mice infected with the *STE11/P*_*tet-off*_*-STE11*^*ΔN467*^ strain exhibited an average of 2.08x10^5^ CFU g^-1^ kidney (Fig 2B). Thus, there was only an ~2-fold difference between them (p<0.0001). This is a >15-fold difference in fungal burden trends when compared to the same infection conditions in immunocompetent mice (Figs 1A and 2B). Furthermore, survival analysis revealed that mice infected with either strain succumbed to infection at a similar rate (Fig 2C), abolishing the prolonged survival previously reported [25]. Collectively, this demonstrates that the host immune system is required for virulence attenuation caused by overexpression of the *STE11*^*ΔN467*^ allele.

It is important to note that the ~2-fold decrease in fungal burden in the immunocompromised mice (Fig 2A) is consistent with an ~1.68-fold difference that was observed in immunocompetent mice that received doxycycline and were infected with the *STE11/P*_*tet-off*_*-STE11*^*ΔN467*^ strain compared to wild-type (Fig 1A). This consistent difference might be due to other factors unrelated to the host immune system, such as a modest fitness defect.

Although immunosuppression via cyclophosphamide treatment was able to show a reliance on the host immune system for virulence attenuation during infection with an unmasked hyperactive *STE11*^*ΔN467*^ mutant, it was still unclear what primary immune cells were contributing to the enhanced fungal clearance. This is due to the broad depletion of different host immune cells induced by cyclophosphamide treatment (S2 Fig), including both circulatory monocytes and neutrophils. Neutrophils have been shown to play a major role in fungal clearance and harbor multiple receptors, such as dectin-1, compliment receptor 3 (CR3) and EphA2, that are capable of recognizing exposed ß(1,3)-glucan to facilitate an immune response [10,12,13,28]. We therefore hypothesized that neutrophils would also play a major role in the clearance of an unmasked *STE11/P*_*tet-off*_*-STE11*^*ΔN467*^ strain. To test this, neutropenia was specifically induced via recurring injections with an anti-Ly6G antibody (1A8) that is specific for depleting host neutrophils (Figs 2D and S3) [29,30]. Mice receiving either the 1A8 treatment or a PBS control injection were intravenously infected with 1x10^4^ CFU/mouse of either the wild-type or the *STE11/P*_*tet-off*_*-STE11*^*ΔN467*^ strain and kidney fungal burden was assessed 4 d.p.i.. Control mice that received the PBS drug vehicle control showed very little detectable fungal burden during infection with either strain (S4 Fig). However, upon 1A8 depletion, mice that did not receive doxycycline and were infected with the wild-type showed an average fungal burden of 6.63x10^5^ CFU g^-1^ kidney, while mice infected with *STE11/P*_*tet-off*_*-STE11*^*ΔN467*^ exhibited an average of 1.08x10^5^ CFU g^-1^ kidney (Fig 2E). Thus, there was an ~6-fold difference in fungal burden between them (p<0.0001). This shows that neutrophils are required for the large(~33-fold) decrease in fungal burden observed in Fig 1A. However, the depletion of neutrophils alone did not restore fungal burden as well as cyclophosphamide treatment (Fig 2B), suggesting that loss of additional immune cell types is needed to restore clearance of the unmasked fungi to the levels observed during cyclophosphamide-induced immunosuppression.

### Hyperactive *STE11*^*ΔN467*^ expression induces altered exposure of both ß(1,3)-glucan and chitin in yeast cells

**Fig 3:**
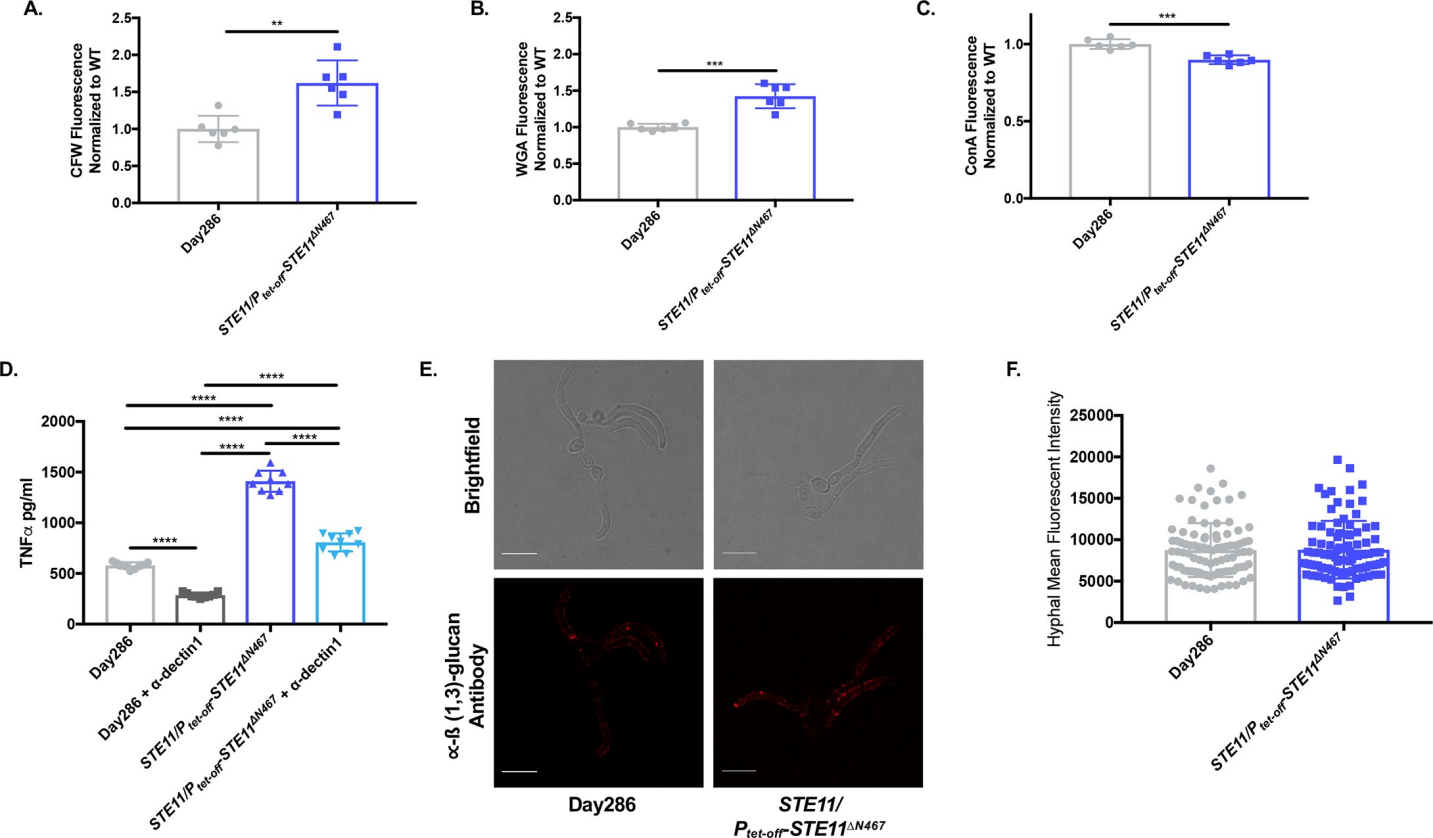
Hyperactive *STE11*^*ΔN467*^ expression induces changes in cell wall components of yeast, but not hyphal cells of *C*. *albicans*. (A-C) Overnight cultures of Day286 wild-type and *STE11/P*_*tet-off*_*-STE11*^*ΔN467*^ cells grown in YPD without doxycycline were stained with calcofluor white (CFW), fluorescein conjugated wheat germ agglutinin (WGA) and Alexa Fluor 647 conjugated concanavalin A (ConA) to assess total chitin, surface exposed chitin, and mannan, respectively. Three biological replicates with 2 technical replicates were run for each sample. (A) CFW staining, (B) WGA staining and (C) ConA staining. (**p<0.005, ***p<0.0005, by student’s t-test). (D) The impact that dectin-1 recognition of exposed ß(1,3)-glucan has on macrophage activation was assessed via antibody blocking using an anti-dectin-1 monoclonal neutralizing antibody. RAW264.7 macrophages and UV-inactivated Day286 wild-type or *STE11/P*_*tet-off*_*-STE11*^*ΔN467*^ cells were co-incubated for 4 hours at a multiplicity of infection (M.O.I.) of 10. Prior to infection, macrophages were either pretreated with 5ug/ml of the anti-dectin-1 neutralizing antibody or a corresponding volume of tissue culture media lacking the antibody. Following co-incubation with yeast cells, supernatants were collected and filtered through a 0.22μm filter and secreted TNFα levels were determined via an ELISA. (****p<0.0001, by one-way ANOVA with Tukey’s post hoc analysis). (E-F) Hyphal cells of the Day286 wild-type and the *STE11/P*_*tet-off*_*-STE11*^*ΔN467*^ strain were stained with anti-ß(1,3)-glucan antibody and a Cy3-conjugated secondary antibody for microscopic analysis of hyphal ß(1,3)-glucan unmasking. (E) Representative confocal microscopy image of stained hyphal cells. The scale bar indicates 10μm. (F) Quantification of hyphal immunofluorescence via ImageJ analysis (n = 3 independent replicates for each strain with 30 hyphal cells quantified per replicate)(p = 0.9258, by student’s t-test).

Hyperactivate *STE11*^*ΔN467*^ attenuates virulence in a manner that is dependent on the host immune system, and this correlates with increased exposure of ß(1,3)-glucan on yeast cells *in vitro* [25,26]. However, ß(1,3)-glucan is only one of several PAMPs in the fungal cell wall, [25,31–35] and it is possible that the exposure (or total levels) of other immunogenic cell wall epitopes, such as chitin and mannan, are altered by *STE11*^*ΔN467*^ expression. To address this, the wild-type and *STE11/P*_*tet-off*_*-STE11*^*ΔN467*^ strains were stained with calcofluor white, wheat germ agglutinin and concanavalin A to assess the levels of total chitin, exposed chitin and mannan, respectively. Staining and subsequent flow cytometry in conditions that overexpress *STE11*^*ΔN467*^ (no doxycycline) revealed a significant increase in both total and exposed chitin levels within the cell wall of the *STE11/P*_*tet-off*_*-STE11*^*ΔN467*^ mutant when compared to the wild-type (Figs 3A and 3B and S5). Furthermore, this was accompanied by a modest decrease in concanavalin A binding in the *STE11/P*_*tet-off*_*-STE11*^*ΔN467*^ strain, suggesting a slight reduction in mannan levels in the outer cell wall (Fig 3C). Thus, hyperactivation of the Cek1 MAPK pathway induces robust changes in both the exposure and total levels of immunogenic cell wall epitopes in addition to ß(1,3)-glucan that may contribute to the observed virulence attenuation.

We have previously reported that co-incubation of RAW264.7 macrophages with the *STE11/P*_*tet-off*_*-STE11*^*ΔN467*^ mutant causes a significant increase in the levels of TNFα production when compared to the wild-type [25]. As our results in Fig 3A–3C show altered levels of multiple immunogenic epitopes in the cell wall, we next sought to assess the sole impact that ß(1,3)-glucan unmasking has on the previously observed *in vitro* immune activation of macrophages. Thus, we performed an antibody blocking assay using an anti-dectin-1 antibody to block the dectin-1 receptor, a prominent ß(1,3)-glucan receptor, on RAW264.7 macrophages and subsequently assessed the levels of TNFα production following co-incubation with UV-inactivated wild-type or *STE11/P*_*tet-off*_*-STE11*^*ΔN467*^ cells. In accordance with our previous findings, co-incubation of RAW264.7 macrophages with *STE11/P*_*tet-off*_*-STE11*^*ΔN467*^ cells caused a significant increase in TNFα production when compared to cells co-incubated with the wild-type control (p<0.0001) (Fig 3D). However, in macrophage samples pretreated with the anti-dectin-1 antibody there was a significant reduction in the levels of TNFα production upon co-incubation with the *STE11/P*_*tet-off*_*-STE11*^*ΔN467*^ strain (p<0.0001). Yet, it is important to note that the observed reduction was not a complete restoration to wild-type levels of TNFα production. This shows that although dectin-1 mediated immune signaling is required for TNFα production following co-incubation with unmasked hyperactive *STE11*^*ΔN467*^ cells, there are also likely independent stimuli aside from exposed ß(1,3)-glucan that are contributing to the increased immune activation induced by the *STE11/P*_*tet-off*_*-STE11*^*ΔN467*^ mutant. Thus, although this experiment does not rule out an important role for the other PAMPs, it does show that ß(1,3)-glucan exposure is required for hyperactive *STE11*^*ΔN467*^-induced TNFα production by macrophages.

### Hyperactive *STE11*^*ΔN467*^ induced ß(1,3)-glucan unmasking is specific to the yeast morphology of *C*. *albicans*

It is clear that hyperactive *STE11*^*ΔN467*^ expression causes ß(1,3)-glucan unmasking in an immunogically relevant manner in yeast cells (Fig 3). However, during systemic infection there is a heterogenous population of both yeast and hyphal cells that are necessary for *C*. *albicans* pathogenesis [36,37]. Therefore, we tested whether hyperactive *STE11*^*ΔN467*^ expression caused ß(1,3)-glucan exposure on hyphal cells as well. To achieve this, hyphal formation was induced in wild-type and *STE11/P*_*tet-off*_*-STE11*^*ΔN467*^ strains, and ß(1,3)-glucan exposure was measured by immunofluorescence microscopy with anti-ß(1,3)-glucan antibody staining (Fig 3E and 3F). Quantification of staining using ImageJ showed no significant difference in the levels of ß(1,3)-glucan exposure between the wild-type and *STE11/P*_*tet-off*_*-STE11*^*ΔN467*^ strains (Fig 3F), demonstrating that the impact that hyperactivate *STE11*^*ΔN467*^ expression has on unmasking is specific to the yeast morphology of *C*. *albicans*.

### Hyperactive *STE11*^*ΔN467*^ induces ß(1,3)-glucan unmasking through the canonical Ste11-Hst7-Cek1 signal cascade

**Fig 4:**
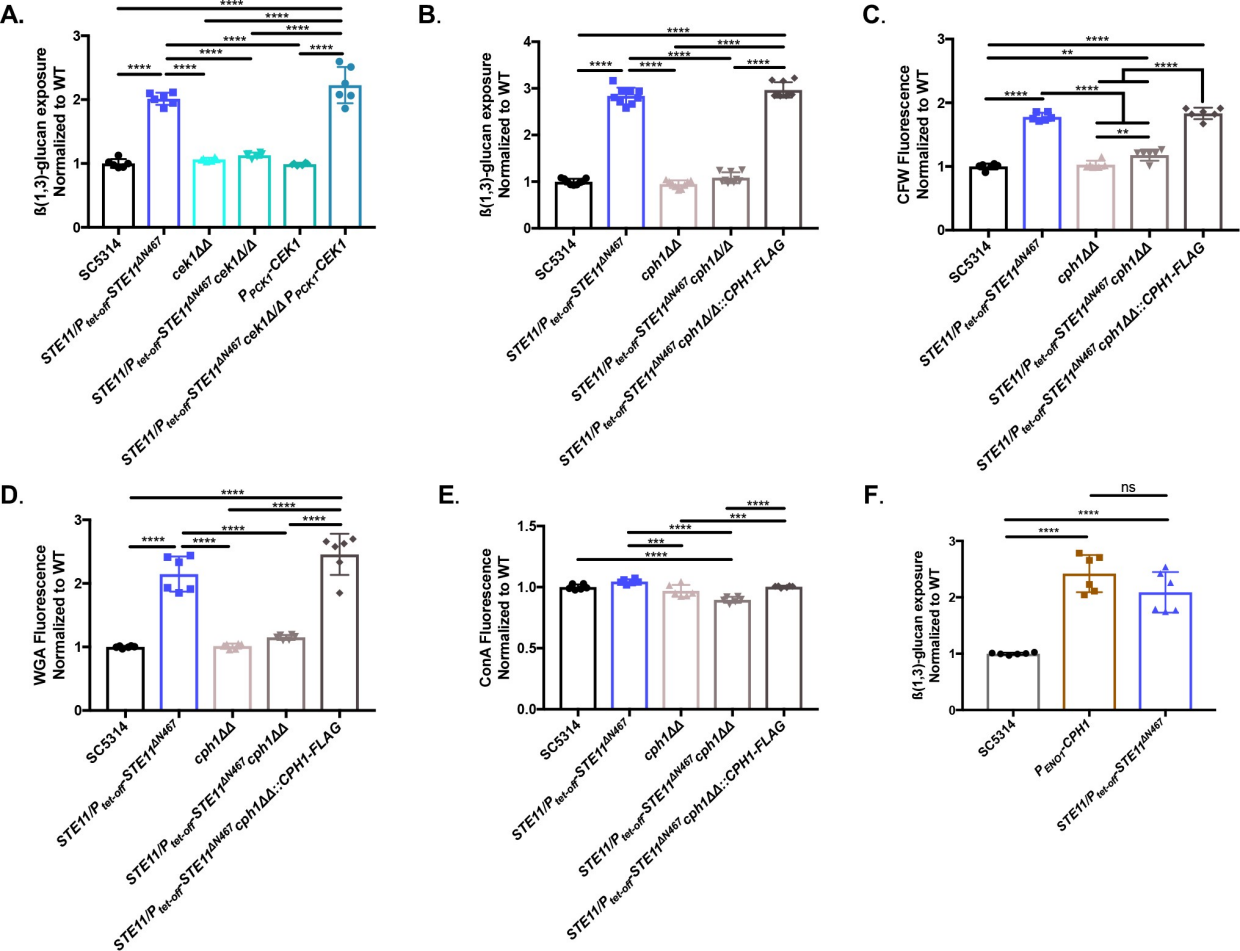
Disruption of downstream components in the Cek1 MAPK pathway blocks ß(1,3)-glucan unmasking by hyperactive Ste11^ΔN467^. (A-B) Overnight cultures of cells were stained with anti-ß(1,3)-glucan antibody and a phycoerythrin-conjugated secondary antibody followed by flow cytometry analysis to assess the levels of ß(1,3)-glucan exposure. (A) ß(1,3)-glucan unmasking of *cek1ΔΔ* mutants. (B) ß(1,3)-glucan unmasking of *cph1ΔΔ* mutants. (C-E) Overnight cultures of cells were stained with calcofluor white (CFW), fluorescein conjugated wheat germ agglutinin (WGA) and Alexa Fluor 647 conjugated concanavalin A (ConA) to assess total chitin, surface exposed chitin, and mannan levels, respectively, of *cph1ΔΔ* mutants. (C) CFW staining, (D) WGA staining and (E) ConA staining. (F) Overnight cultures were stained to assess ß(1,3)-glucan unmasking of the *P*_*ENO1*_*-CPH1* overexpression mutant. For all stains, three biological replicates with 2–3 technical replicates were analyzed by flow cytometry for each sample. (**p<0.01, ***p<0.001, ****p<0.0001, by one-way ANOVA with Tukey’s post hoc analysis).

Since hyperactivation of *STE11*^*ΔN467*^ induces changes in the levels of multiple immunogenic epitopes within the cell wall, we next sought to identify the mechanism driving these changes. We had previously shown that Ste11^*Δ*N467^ causes downstream activation of the MAPK Cek1 [25], which led us to hypothesize that signal transduction is propagated through this protein. To test this, we created a *cek1ΔΔ* mutation in a CRISPR-Cas9-competent wild-type strain (SC5314 *LEU2/leu2Δ*) and a *STE11/P*_*tet-off*_*-STE11*^*ΔN467*^ mutant in the same wild-type background to assess the impact of Cek1 on unmasking. Immunofluorescent staining using an anti-ß(1,3)-glucan antibody and subsequent flow cytometry revealed that a *cek1ΔΔ* mutation in the wild-type background did not impact unmasking (Fig 4A). However, a *STE11/P*_*tet-off*_*-STE11*^*ΔN467*^*cek1ΔΔ* double mutant successfully blocked the unmasking induced by hyperactive Ste11^ΔN467^ alone, which was restored upon reintroduction of a functional *CEK1* gene under the control of the constitutive phosphoenolpyruvate carboxykinase promoter *(P*_*PCK1*_*-CEK1)*. Therefore, hyperactive Ste11^ΔN467^ induces unmasking through the MAPK Cek1.

Interestingly, while the data presented supports the role of Cek1 in mediating hyperactive Ste11^ΔN467^ induced unmasking, our findings are in disagreement with other studies in which *CEK1* was deleted. Specifically, it has been shown that a homozygous deletion of *CEK1* in the CAI4 wild-type background is sufficient to induce unmasking on its own [38,39]. Although the *cek1ΔΔ* mutant used in our study did not induce this same phenotype, it is important to note that it was made in the SC5314 strain background, rather than CAI4. Thus, to determine if the observed differences associated with a *cek1ΔΔ* mutation were due to differences in the wild-type strains used, we created a *cek1ΔΔ* mutant in the CAI4 background using CRISPR-Cas9. Consistent with the phenotype observed during disruption in the SC5314 background, *CEK1* deletion in the CAI4 laboratory strain did not impact unmasking (S6 Fig), demonstrating that strain variability (of the strains in our possession) does not account for the reported differences in unmasking caused by *cek1ΔΔ*.

Our data implicate Cek1 as the downstream MAPK mediating hyperactive Ste11^ΔN467^-induced unmasking, yet it was still unclear if signal transduction to Cek1 was mediated through the canonical Ste11-Hst7-Cek1 signal cascade. To address this, we created a *hst7ΔΔ* mutation in both the wild-type and *STE11/P*_*tet-off*_*-STE11*^*ΔN467*^ strains. Similar to our observations using *cek1ΔΔ* mutants, deletion of *HST7* in the *STE11/P*_*tet-off*_*-STE11*^*ΔN467*^ strain (*STE11/P*_*tet-off*_*-STE11*^*ΔN467*^*hst7ΔΔ*) completely restored unmasking to wild-type levels (S7 Fig). Thus, hyperactive Ste11^ΔN467^-induced unmasking is mediated via the canonical Ste11-Hst7-Cek1 signal transduction pathway.

### Cek1 induces ß(1,3)-glucan unmasking through the transcription factor Cph1

Cek1 propagates signal transduction through two main transcription factors, Cph1 and Ace2 [32,40–43]. Although our findings have shown that Cek1 is acting to cause ß(1,3)-glucan unmasking, it was not clear which downstream transcription factor is required, as both Cph1 and Ace2 activation impact cell wall architecture [24,32,42,44–46]. Our prior RNA sequencing results revealed that *CPH1*, but not *ACE2*, transcript levels are upregulated in a *STE11/P*_*tet-off*_*-STE11*^*ΔN467*^ mutant [25]. Therefore, we hypothesized that Cek1 mediates Ste11^ΔN467^-induced unmasking through Cph1. To this end, we created a *cph1ΔΔ* mutation in both the wild-type and *STE11/P*_*tet-off*_*-STE11*^*ΔN467*^ strains to epistatically test the role of Cph1 in unmasking. Immunofluorescent staining for exposed ß(1,3)-glucan and subsequent flow cytometry revealed that a *STE11/P*_*tet-off*_*-STE11*^*ΔN467*^*cph1ΔΔ* double mutant returns ß(1,3)-glucan exposure to wild-type levels, and that unmasking is restored in the double mutant upon complementation with a C-terminally FLAG-tagged *CPH1* gene (Fig 4B). Furthermore, staining for other immunogenic epitopes in the cell wall revealed that the *STE11/P*_*tet-off*_*-STE11*^*ΔN467*^*cph1ΔΔ* mutant also restored calcofluor white and wheat germ agglutinin binding to wild-type levels (Fig 4C and 4D), demonstrating that Cph1 is the transcription factor mediating changes for both the ß(1,3)-glucan and chitin exposure observed during *STE11*^*ΔN467*^ expression. Interestingly, concanavalin A staining of a *STE11/P*_*tet-off*_*-STE11*^*ΔN467*^ mutant in the SC5314 wild-type background exhibited no changes in mannan levels compared to the wild-type (Fig 4E), but did show a modest reduction upon *CPH1* deletion. Altogether, our data demonstrate that hyperactive Ste11^*Δ*N467^ functions through Cph1 to mediate both chitin and ß(1,3)-glucan exposure, but it is not clearly involved in inducing changes in cell wall mannan levels.

As it appears that Ste11^ΔN467^-induced hyperactivation of Cph1 is sufficient to induce unmasking, we further hypothesized that overexpression of *CPH1* on its own would increase ß(1,3)-glucan exposure. To this end, we created a *CPH1* overexpression strain by placing a third allele of *CPH1* under the control of the constitutive enolase (*ENO1*) promoter (*P*_*ENO1*_*-CPH1*). Immunofluorescent staining revealed that the *P*_*ENO1*_*-CPH1* mutant exhibited a significant increase in ß(1,3)-glucan exposure when compared to the wild-type (Fig 4F) (p<0.0001) and had similar levels of unmasking to the *STE11/P*_*tet-off*_*-STE11*^*ΔN467*^ mutant (p = 0.1362). Therefore, it appears that hyperactivation of *CPH1* alone is sufficient to induce ß(1,3)-glucan unmasking.

### Deletion of *CPH1* in a hyperactive *STE11*^*ΔN467*^ background blocks macrophage immune activation and restores virulence during systemic infection in mice

**Fig 5:**
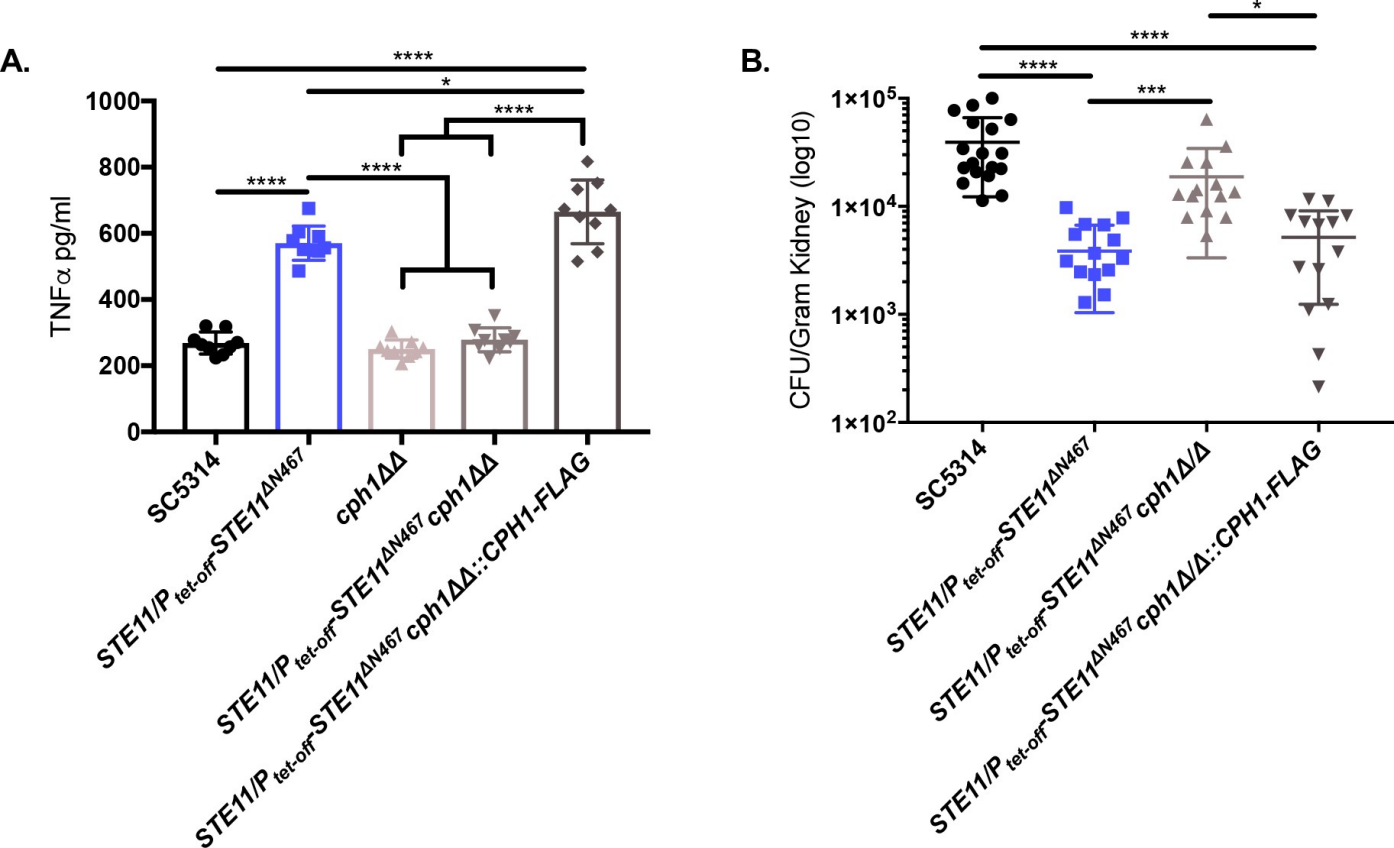
*CPH1* deletion reduces Ste11^ΔN467^ induced TNFα secretion by macrophages and partially restores virulence during systemic infection in mice. (A) TNFα secretion by RAW264.7 macrophages. RAW264.7 murine macrophages and UV-inactivated *C*. *albicans* strains were co-incubated for 4 hours at a multiplicity of infection (M.O.I.) of 10. Culture supernatant was subsequently filtered through a 0.22μm filter and assessed via ELISA analysis to determine secreted TNFα levels. (*p = 0.0063, ****p<0.0001, by one-way ANOVA with Tukey’s post hoc analysis)(n = 3 biological replicates with 3 technical replicates for each sample). (B) Kidney fungal burden 4 d.p.i. during infection with *cph1ΔΔ* mutants. ICR mice were intravenously injected with 1x10^6^ cells of *C*. *albicans* wild-type (SC5314), *STE11/P*_*tet-off*_*-STE11*^*ΔN467*^, *STE11/P*_*tet-off*_*-STE11*^*ΔN467*^*cph1ΔΔ* or the *STE11/P*_*tet-off*_*-STE11*^*ΔN467*^*cph1ΔΔ*::*CPH1-Flag* strain and kidneys were harvested 4 d.p.i to assess fungal burden. (n = 7–8 mice)(*p<0.05, ***p<0.001, ****p<0.0001, by Kruskal-Wallis test with Dunn’s multiple comparisons post-hoc analysis.).

Hyperactive *STE11*^*ΔN467*^ expression is sufficient to induce unmasking of ß(1,3)-glucan and chitin (Fig 4), increase TNFα production by RAW264.7 macrophages *in vitro* (Fig 3D), and attenuate virulence during a systemic infection model in mice (Fig 1A). As our data have shown that a *cph1ΔΔ* mutation in the *STE11/P*_*tet-off*_*-STE11*^*ΔN467*^ background is capable of blocking hyperactive Ste11^*Δ*N467^ induced exposure of both ß(1,3)-glucan and chitin, we further hypothesized that it will also reduce macrophage immune activation *in vitro*. To test this, *CPH1* knockout mutants were UV-irradiated and co-incubated with RAW264.7 macrophages, and TNFα secretion was assessed by ELISA (Fig 5A). This experiment revealed that introduction of the *cph1*ΔΔ mutation into the *STE11/P*_*tet-off*_*-STE11*^*ΔN467*^ strain reduced TNFα secretion from murine macrophages to wild-type levels.

These data suggested that loss of *CPH1* would also be sufficient to restore virulence of the *STE11/P*_*tet-off*_*-STE11*^*ΔN467*^ strain during systemic infections in mice. To test this, mice were intravenously injected with 1x10^6^ CFU of the wild-type, *STE11/P*_*tet-off*_*-STE11*^*ΔN467*^, *STE11/P*_*tet-off*_*-STE11*^*ΔN467*^*cph1ΔΔ* or *STE11/P*_*tet-off*_*-STE11*^*ΔN467*^*cph1ΔΔ*::*CPH1-Flag* reintegrate strains. Kidneys were then harvested 4 d.p.i. to assess fungal burden. As shown in Fig 5B, mice infected with the *STE11/P*_*tet-off*_*-STE11*^*ΔN467*^*cph1ΔΔ* double mutant had a significant increase in fungal burden toward wild-type levels when compared to the *STE11/P*_*tet-off*_*-STE11*^*ΔN467*^ parent strain (p = 0.0009). Furthermore, fungal burden attenuation was restored upon complementation with a C-terminally flag-tagged *CPH1* allele. Therefore, *CPH1* deletion in a hyperactive *STE11*^*ΔN467*^ background is capable of restoring fungal burden toward wild-type levels during a systemic infection in mice.

### Cph1 functions in a positive feedback loop to increase hyperactivation of the upstream MAPK Cek1

**Fig 6:**
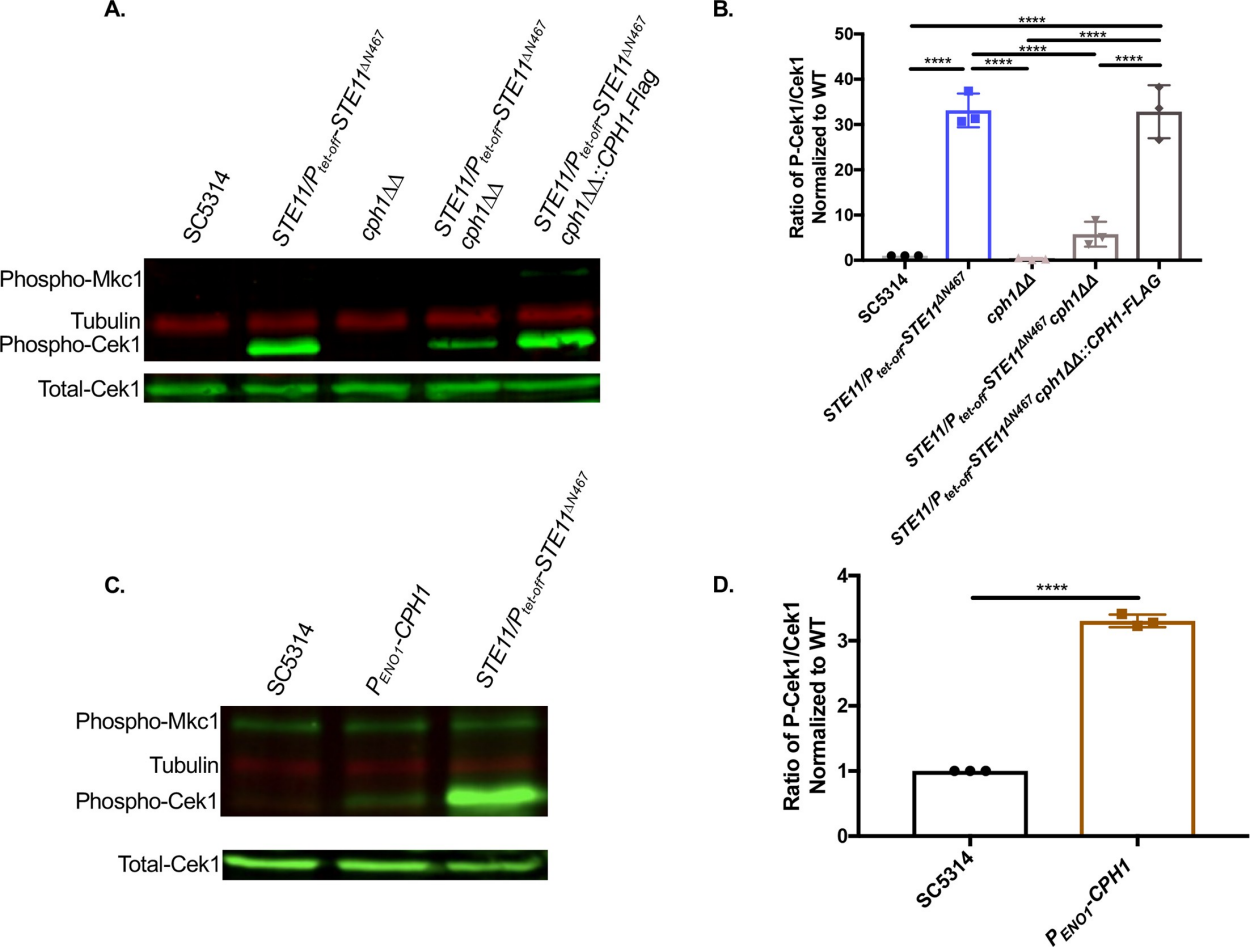
Cph1 mediates activation of its upstream MAPK, Cek1. Proteins were harvested from cells grown to mid-log phase for western blot analysis. Membranes were blotted using an anti-P44/42 antibody for phosphorylated Cek1 detection and an anti-Cek1 antibody for total Cek1 detection. (A) Western blot showing active and total Cek1 levels in *cph1*ΔΔ deletion mutants. (B) Fold change relative to the wild-type in Phosphorylated-Cek1 to total Cek1 levels for all *CPH1* deletion mutants. (n = 3 biological replicates assessed on 3 separate western blots) (****p<0.0001, by one-way ANOVA with Tukey’s post hoc analysis). (C) Western blot showing active and total Cek1 levels for the *P*_*ENO1*_*-CPH1* overexpression mutant. (D) Fold change relative to the wild-type in Phosphorylated-Cek1 to total Cek1 levels for the *P*_*ENO1*_*-CPH1* overexpression mutant. (n = 3 biological replicates assessed on 3 separate western blots) (****p<0.0001, by students t-test).

RNA sequencing experiments that compared transcriptomes of the *STE11/P*_*tet-off*_*-STE11*^*ΔN467*^ strain and the wild-type control revealed that during *STE11*^*ΔN467*^ expression there was increased expression of Cph1, Cek1, and some components of the Cek1 MAPK pathway that sit upstream of the transcription factor and kinase [25]. Specifically, the cell wall sensors *OPY2* and *DFI1*, both of which have been shown to impact Cek1 activation [47–49], were upregulated. As our results indicate that Cph1 is driving unmasking of both ß(1,3)-glucan and chitin (Fig 4B and 4D), the increased expression of these upstream components of the signaling pathway, and increased expression of *CPH1* itself, suggests the possibility that Cph1 induces a positive feedback loop to amplify its own transcriptional response. This is further supported by ChIP-chip data showing that Cph1 binds directly to the promoters of *CEK1* and *OPY2*, as well as to its own promoter [50]. We therefore assessed the impact that a *cph1ΔΔ* disruption has on activation of its own upstream MAPK, Cek1. To do this, we measured the levels of the phosphorylated (active) form of Cek1 in the wild-type, *STE11/P*_*tet-off*_*-STE11*^*ΔN467*^, *cph1ΔΔ*, *STE11/P*_*tet-off*_*-STE11*^*ΔN467*^*cph1ΔΔ* and *STE11/P*_*tet-off*_*-STE11*^*ΔN467*^*cph1ΔΔ*::*CPH1-Flag* reintegrate strains. As shown in Fig 6A and 6B, activation of Cek1 was ~33-fold higher in the *STE11/P*_*tet-off*_*-STE11*^*ΔN467*^ strain during *STE11*^*ΔN467*^ induction when compared to the wild-type. However, phosphorylated Cek1 levels were significantly reduced in the *STE11/P*_*tet-off*_*-STE11*^*ΔN467*^*cph1ΔΔ* double mutant (~5.77-fold over wild-type) compared to its *STE11/P*_*tet-off*_*-STE11*^*ΔN467*^ parent strain (p<0.0001). Therefore, it appears that expression of the downstream transcription factor *CPH1* is necessary for full hyperactivation of Cek1 in the *STE11/P*_*tet-off*_*-STE11*^*ΔN467*^ strain and suggests that a loop is necessary for the full activation of the transcriptional circuit. It was of interest to note that the level of total Cek1 did not change, indicating that the increase in phosphorylated Cek1 was not simply a result of increased levels of the Cek1 protein overall. This is further supported by the observation that *CPH1* overexpression on its own is also sufficient for impacting the activation of its upstream MAPK, Cek1, but does not change the total levels of Cek1 observed. Western blot analysis of phosphorylated-Cek1 levels demonstrated a 3.30-fold increase in Cek1 activation in the *P*_*ENO1*_*-CPH1* mutant compared to the wild-type (Fig 6C and 6D) (p<0.0001). Thus, it appears that Cph1 induces a positive feedback loop to amplify signal transduction and regulate the magnitude of its corresponding phenotypic modifications.

### The cell wall sensor Dfi1 is needed for Cph1-mediated ß(1,3)-glucan unmasking

**Fig 7:**
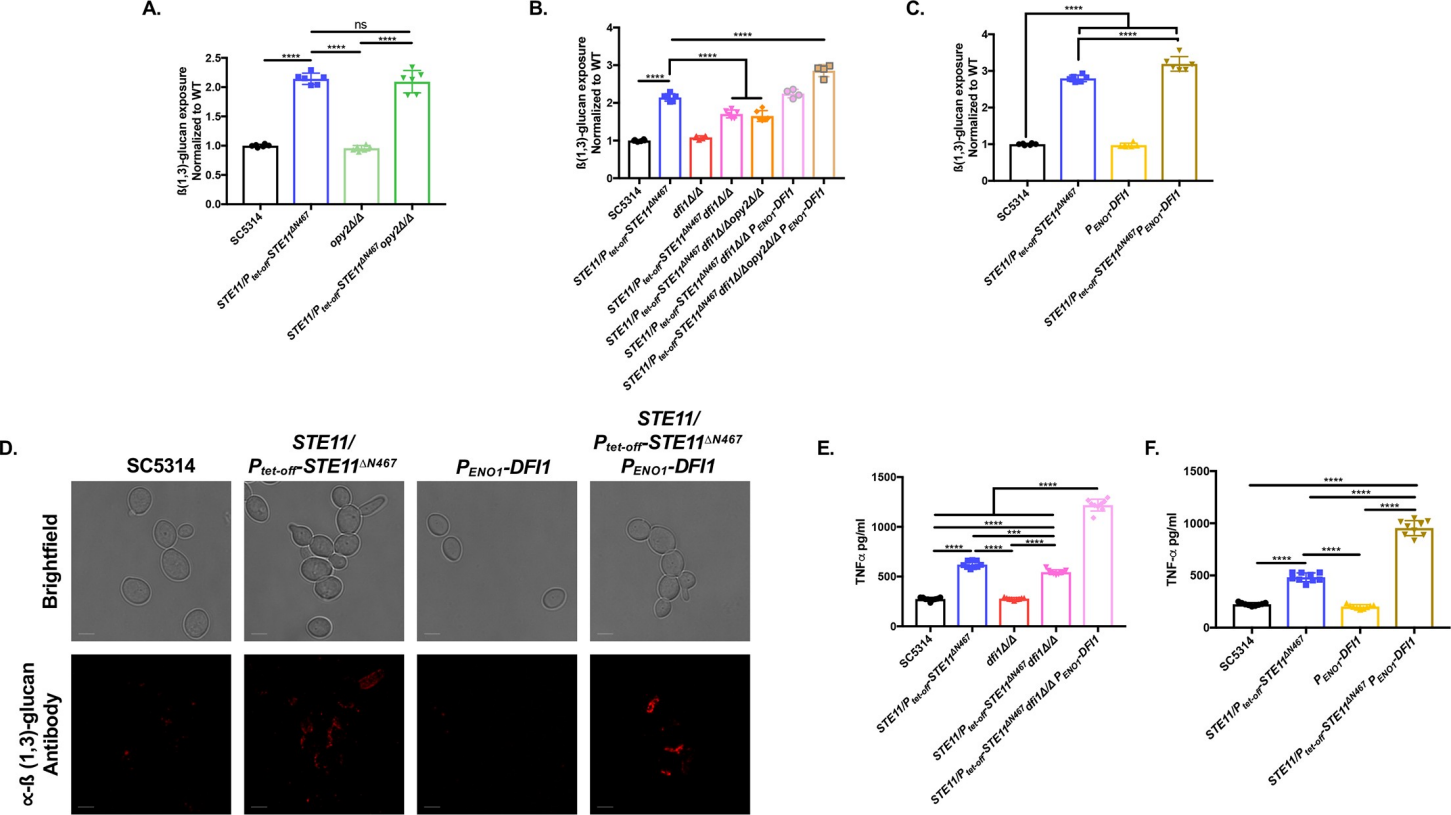
*DFI1* expression levels partially mediate ß(1,3)-glucan unmasking and macrophage TNFα secretion induced by hyperactive *STE11*^*ΔN467*^ expression. (A-D) Overnight cultures of cells were stained with anti-ß(1,3)-glucan antibody and a phycoerythrin-conjugated secondary antibody for flow cytometry analysis or a Cy3-conjugated secondary antibody for confocal microscopy to assess the levels of ß(1,3)-glucan exposure. (A) ß(1,3)-glucan unmasking of *opy2ΔΔ* mutants. (B) ß(1,3)-glucan unmasking of *dfi1ΔΔ* mutants. (C) ß(1,3)-glucan unmasking of *P*_*ENO1*_*-DFI1* overexpression mutants. (****p<0.0001, by one-way ANOVA with Tukey’s post hoc analysis)(n = 3 biological replicates with 2–3 technical replicates were run for each sample.). (D) Representative microscopy images of ß(1,3)-glucan exposure of *DFI1* overexpression mutants. The scale bar indicates 10μm. (E-F) The impact of altered *DFI1* expression levels on macrophage TNFα secretion was assessed via co-incubation of RAW264.7 murine macrophages with UV-inactivated *DFI1* mutants for 4 hours at a multiplicity of infection (M.O.I.) of 10. Culture supernatant was subsequently filtered through a 0.22um filter and assessed via ELISA analysis to determine secreted TNFα levels. (E) TNFα levels during co-incubation with RAW264.7 and *dfi1ΔΔ* mutants. (F) TNFα levels during co-incubation with RAW264.7 and *P*_*ENO1*_*-DFI1* overexpression mutants. (***p<0.0005, ****p<0.0001, by one-way ANOVA with Tukey’s post hoc analysis) (n = 3 biological replicates with 3 technical replicates for each sample).

Cph1 acts in a feedback loop that amplifies activation of Cek1. Our previous RNA sequencing results showed increased transcript levels of two cell wall sensors that have been reported to impact Cek1 activation, *DFI1* and *OPY2*, during hyperactive *STE11*^*ΔN467*^ expression [25]. We therefore hypothesized that these cell wall sensors are also needed for full loop stimulation during Cph1 activation. To address this, we created individual *dfi1ΔΔ* and *opy2ΔΔ* mutants in both the wild-type and *STE11/P*_*tet-off*_*-STE11*^*ΔN467*^ strains. Combinatorial cell wall sensor mutants in both strain backgrounds were also created to account for any redundancy in signal transduction through the pathway. Subsequent immunofluorescent staining and flow cytometry revealed that a *STE11/P*_*tet-off*_*-STE11*^*ΔN467*^*opy2ΔΔ* double mutant had no impact on unmasking when compared to *STE11/P*_*tet-off*_*-STE11*^*ΔN467*^ (Fig 7A) (p = 0.8607). However, a *STE11/P*_*tet-off*_*-STE11*^*ΔN467*^*dfi1ΔΔ* double mutant was partially suppressed for unmasking compared to the *STE11/P*_*tet-off*_*-STE11*^*ΔN467*^ strain (Fig 7B) (p<0.0001). Furthermore, a *STE11/P*_*tet-off*_*-STE11*^*ΔN467*^*dfi1ΔΔopy2ΔΔ* triple mutant was not further reduced for unmasking when compared to the *STE11/P*_*tet-off*_*-STE11*^*ΔN467*^*dfi1ΔΔ* double mutant (p = 0.9684), demonstrating that these two cell wall sensors do not play redundant or additive roles in Cph1 mediated feedback into the Cek1 MAPK pathway.

Interestingly, during reintroduction of *DFI1* under the control of the constitutive enolase promoter (*P*_*ENO1*_*-DFI1*) into the *STE11/P*_*tet-off*_*-STE11*^*ΔN467*^*dfi1ΔΔopy2ΔΔ* triple mutant, we observed an increase in ß(1,3)-glucan unmasking when compared to *STE11/P*_*tet-off*_*-STE11*^*ΔN467*^ alone (Fig 7B). Thus, we were interested in determining the impact that increased *DFI1* expression may have on unmasking during Cph1 hyperactivation. To this end, we induced *DFI1* overexpression by placing it under the control of the constitutive enolase promoter (*P*_*ENO1*_*-DFI1*) in both the wild-type and *STE11/P*_*tet-off*_*-STE11*^*ΔN467*^ background strains. In doing so, these mutants now harbored three alleles of *DFI1* in their genome (both wild-type alleles and the *P*_*ENO1*_*-DFI1* allele at the enolase locus). Immunofluorescent staining and flow cytometry of these mutants revealed that overexpression of *DFI1* on its own in the wild-type background is insufficient to induce unmasking (Fig 7C)(p = 0.9771). However, *DFI1* overexpression in the *STE11/P*_*tet-off*_*-STE11*^*ΔN467*^ parent strain, when hyperactivation of the Cek1 MAPK pathway is induced, further increased the observed unmasking when compared to the *STE11/P*_*tet-off*_*-STE11*^*ΔN467*^ mutant that only harbors the wild-type alleles of *DFI1* (p<0.0001). It is important to note that we have previously shown that Ste11^ΔN467^-induced unmasking occurs within cells in the yeast morphology (Fig 3F) [26], but a *dfi1ΔΔ* mutation has also been previously shown to impact invasion in embedded agar, a process that is largely mediated by hyphal formation [48,49]. Therefore, it was important to assess whether overexpression of *DFI1* under stimulating conditions was inducing a morphological transition into hyphae that may account for the observed increase in unmasking. Immunofluorescent staining and subsequent confocal microscopy revealed that this is not the case, and that unmasking is occurring within the yeast population (Fig 7D). Thus, it appears that the level of Dfi1 in the cell wall of *C*. *albicans* yeast cells can impact the subsequent ß(1,3)-glucan unmasking induced by activated Cph1.

### *DFI1* expression levels impact macrophage TNFα production *in vitro*

Due to the observed impact that *DFI1* expression has on Ste11^ΔN467^-induced unmasking, we next wished to assess its impact on murine macrophage immune activation. Co-incubation of RAW264.7 macrophages and *C*. *albicans DFI1* mutants showed that, as with unmasking, a *STE11/P*_*tet-off*_*-STE11*^*ΔN467*^*dfi1ΔΔ* double mutant was able to partially reduce TNFα production toward wild-type levels (Fig 7E). Furthermore, overexpression of *DFI1* on a third allele in the hyperactive *STE11*^*ΔN467*^ background was also sufficient to significantly increase supernatant TNFα levels when compared to its *STE11/P*_*tet-off*_*-STE11*^*ΔN467*^ parent strain (Fig 7F)(p<0.0001). Moreover, in contrast to what was observed for ß(1,3)-glucan staining alone, TNFα-secretion was increased in the *STE11/P*_*tet-off*_*-STE11*^*ΔN467*^ strain when *DFI1* was overexpressed, even when the two native *DFI1* copies were deleted (compare Fig 7B–7E).

### Dfi1 stimulation impacts ß(1,3)-glucan unmasking through an alternative pathway than the Cek1 MAPK pathway

**Fig 8:**
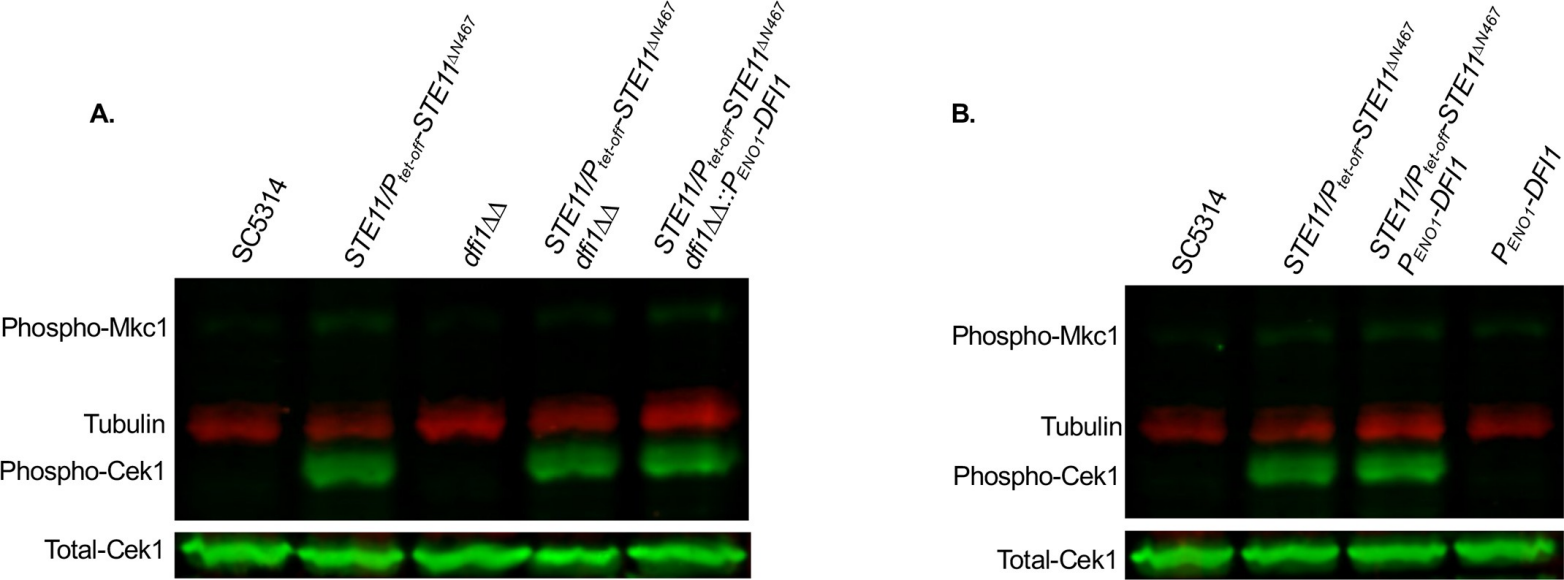
Dfi1 does not mediate Ste11^ΔN467^-induced activation of the MAPK Cek1. Proteins were harvested from cells grown to mid-log phase for western blot analysis. Membranes were blotted using an anti-P44/42 antibody for phosphorylated Cek1 detection and an anti-Cek1 antibody for total Cek1 detection. (A) Western blot showing active and total Cek1 levels in *DFI1* deletion mutants. (B) Western blot showing active and total Cek1 levels in *DFI1* overexpression mutants.

It is clear that Dfi1 impacts ß(1,3)-glucan unmasking and is needed to achieve the full levels of unmasking observed during expression of a hyperactive *STE11*^*ΔN467*^ allele. As Dfi1 has previously been shown to impact activation of the MAPK Cek1, we hypothesized that Dfi1 impacted unmasking levels by serving as a node in the Cph1-mediated positive feedback loop in the Cek1 MAPK pathway. In this model, altered expression levels of *DFI1* would correlate with a corresponding change in the levels of phosphorylated (activated) Cek1. However, neither *DFI1* deletion (Fig 8A) nor *DFI1* overexpression (Fig 8B) mutants showed any impact on Cek1 activation. Thus, it appears that Dfi1 mediates unmasking via a parallel, unidentified signaling pathway that is activated during hyperactive *STE11*^*ΔN467*^ expression.

## Discussion

We have previously shown that overexpression of a single allele of a hyperactive *STE11*^*ΔN467*^ mutant increases ß(1,3)-glucan exposure and attenuates virulence in a mouse model of systemic candidiasis [25,26]. In this report we demonstrate that the virulence attenuation is primarily driven by the host immune response, as cyclophosphamide induced immunosuppression in mice largely negated the decrease in kidney fungal burden 4 days post infection (Figs 1 and 2). Furthermore, we found that while neutrophils play a major role in this fungal clearance, other immune cells appear to be involved in recognizing and responding to infection with an unmasked hyperactive *STE11*^*ΔN467*^ mutant, as 1A8 neutrophil depletion was unable to completely restore fungal colonization levels to those observed during cyclophosphamide induced immunosuppression. Our previous findings also showed that *STE11*^*ΔN467*^ expression leads to increased phosphorylation levels of the MAPK Cek1 [25,26]. Here, we have expanded on these findings and show that Ste11^ΔN467^ functions through the transcription factor *CPH1* to increase both ß(1,3)-glucan and chitin exposure (Fig 4). *CPH1* disruption reduced Ste11^ΔN467^-induced unmasking and TNFα secretion by murine macrophages to wild-type levels, as well as partially restored virulence during systemic infection in mice. Moreover, we have found that Cph1 functions in a positive feedback loop that is necessary to achieve the total activation of Cek1 observed during *STE11*^*ΔN467*^ expression (Fig 6), and that a parallel, as of yet unidentified, pathway is activated via the cell wall sensor Dfi1 to induce the full levels of ß(1,3)-glucan unmasking observed during *STE11*^*ΔN467*^ expression (Figs 7 and 8).

### Regulated activation of the Cek1 MAPK pathway via expression of a hyperactive *STE11*^*ΔN467*^ allele attenuates virulence in a manner that is dependent on the host immune response

Studies in *C*. *albicans* have demonstrated that echinocandin application and certain mutations in genes that impact proper cell wall construction, such as *ENG1* disruption or deletions of the phosphatidylserine synthase gene *CHO1* or the glycosyltransferase gene *KRE5*, induce ß(1,3)-glucan unmasking and attenuate virulence during systemic infection models [5,6,18,51,52]. Furthermore, it has been shown that even different *C*. *albicans* strains vary in the baseline level of unmasking [53]. From these findings a clear correlation between *C*. *albicans* unmasking and immune activation has been established *in vitro* [6,21,51]. This relationship is further supported from studies investigating *H*. capsulatum, where unmasking induced by deletion of the endo-1,3-beta-glucanse *ENG1* was shown to attenuate virulence *in vivo* in a dectin-1 dependent manner [17]. Yet, direct interpretation of the impact of unmasking of *C*. *albicans* cells *in vivo* has largely been confounded by the additional cidal effects of echinocandin application, substantial secondary fitness defects with genetic mutants, such as an ethanolamine auxotrophy in the *cho1ΔΔ* strain [54], and additional factors that are variable between *C*. *albicans* strains. Our results indicate that the *STE11/P*_*tet-off*_*-STE11*^*ΔN467*^ mutant exhibits ß(1,3)-glucan unmasking, increases both total chitin levels and exposure and attenuates virulence in a host immune system dependent manner, without inducing gross secondary fitness defects that impact its ability to colonize the host. This is apparent in the restoration of kidney fungal burden from the observed ~33-fold decrease during systemic infection with the *STE11/P*_*tet-off*_*-STE11*^*ΔN467*^ strain in immunocompetent mice (Fig 1A), to only an ~2-fold difference when compared to the wild-type in cyclophosphamide immunosuppressed mice (Fig 2B). Although a significant difference in fungal burden still exists during cyclophosphamide induced immunosuppression, suggesting a modest fitness defect, the ability of immunodepletion to restore fungal burden to the nearly wild-type levels for the *STE11/P*_*tet-off*_*-STE11*^*ΔN467*^ mutant suggests that the virulence attenuation is primarily host immune system driven. We therefore propose this pathway as an applicable *in vivo* model to analyze how the host immune system recognizes and responds to unmasked *C*. *albicans* cells to mount an immune response.

In application of this model, we have shown that depletion of neutrophils alone is not sufficient to restore fungal burden of an unmasked hyperactive *STE11*^*ΔN467*^ mutant to wild-type levels (Fig 2D). Neutrophil depletion was able to change the ~33-fold decrease in fungal burden caused by hyperactive *STE11*^*ΔN467*^ expression (Fig 1A) to an ~6-fold difference, indicating a major role for these immune cells in clearing the unmasked fungi. However, the inability of neutrophil depletion to restore fungal colonization between these strains to the ~2-fold difference observed during cyclophosphamide treatment suggests that other immune cells are involved in facilitating the enhanced fungal clearance caused by *STE11*^*ΔN467*^ expression. Multiple immune cells, aside from neutrophils, harbor receptors capable of recognizing exposed ß(1,3)-glucan and chitin and initiating an immune response. These cell types include important immune modulators such as monocytes, macrophages, dendritic cells and host epithelial cells [7,14,34,38,55]. Our data have already shown increased immune activation of murine macrophages *in vitro* in response to exposure to unmasked *STE11*^*ΔN467*^ cells (Fig 3D), and it is likely that tissue resident and infiltrating macrophages play a similar role in recognizing and initiating an immune response against unmasked fungal cells within the kidney. It is possible that unmasked fungi are more readily recognized by tissue resident macrophages, which likely phagocytose to both kill and to establish a more rapid and robust cytokine response that facilitates recruitment of other immune cells, like neutrophils, to the site of infection. However, it is thus far unclear if neutrophils also more readily recognize unmasked fungi to increase their killing efficiency, or whether they are simply recruited more quickly to the site of infection by enhanced recognition of unmasked fungi by tissue resident immune cells. Consequently, more work is needed to understand the interplay within the host immune system in responding to systemic infections with unmasked *C*. *albicans* cells.

### Hyperactive Ste11^ΔN467^ functions through the canonical Ste11-Hst7-Cek1 MAPK pathway and the transcription factor Cph1 to induce ß(1,3)-glucan unmasking and increase chitin exposure

In this report we have shown that deletion of either the MAPKK *HST7* or the MAPK *CEK1* in a hyperactive *STE11*^*ΔN467*^ mutant background effectively blocks ß(1,3)-glucan unmasking (Figs 4A and S7). This is expected in our current model, as *STE11*^*ΔN467*^ expression shows a specific hyperactivation of Cek1 itself [26], and downstream regulons under the control of Cek1 have been shown to regulate cell wall architecture and repair [32,42,44,46]. However, our data is in contradiction with results previously published on *CEK1*, where it was shown that a *cek1ΔΔ* mutation on its own results in increased unmasking [38,39]. We have shown that this does not appear to be the consequence of strain variation between studies analyzing *cek1ΔΔ-*induced unmasking, as we were unable to observe increased ß(1,3)-glucan exposure in CIA4 *CEK1* deletion mutants (S6 Fig). However, lab to lab strain variations cannot be ruled out either.

The transcription factor Cph1 has previously been reported to be involved in phenotypic switching that allows mating [42], as well as in filament formation [44]. Here, we have shown that Cph1 causes unmasking downstream of hyperactive Ste11^ΔN467^ (Fig 4B), as well as when overexpressed on its own (Fig 4F). Furthermore, *CPH1* is also required for increased chitin exposure induced by hyperactive *STE11*^*ΔN467*^ expression (Fig 4D). This suggests that altered expression of components within the Cph1 regulon are capable of inducing unmasking and impacting the overall architecture of the yeast cell wall. Cph1 has been shown to regulate multiple components involved in cell wall construction. Some of these include chitin synthase genes [56], as well genes involved in hyphal development [41,56]. Our previous results, based on RNA sequencing and Als3 protein levels, revealed that expression of *STE11*^*ΔN467*^ did not induce a strong yeast to hyphae transition (Fig 3E and 3F), but did show overexpression of several genes involved in regulating cell wall architecture [25,26]. Among these genes, chitin synthases 2 (*CHS2*), 8 (*CHS8*), and 7 (*CHS7*) were both found to be upregulated by *STE11*^*ΔN467*^ expression [25]. Several studies have shown that areas of increased chitin deposition in hyphal cells co-localize with unmasked ß(1,3)-glucan foci [57,58]. As we have observed an increase in both total chitin and exposed chitin within the yeast cells of *C*. *albicans*, it may be possible that Cph1 induces ß(1,3)-glucan unmasking by increased expression of chitin synthase genes that cause a disruption in the normal cell wall architecture of *C*. *albicans*. Alternatively, there have been a number of recent publications showing the impacts that secreted glucanases have on regulating unmasking. For example, the exo-1,3-beta-glucanase Xog1 has been implicated as the mechanism driving ß(1,3)-glucan masking in *C*. *albicans* in response to lactate exposure [19], while the endo-1,3-beta-glucanse Eng1 has recently been shown to predominantly regulate unmasking in *C*. *albicans* yeast cells [18]. With regards to the Cek1 pathway, our previous transcriptomics results have shown that *XOG1* was ~50-fold up-regulated and *ENG1* was ~2-fold down-regulated during hyperactive *STE11*^*ΔN467*^ expression [25]. It is possible that the reduced expression of *ENG1* may play a role in the observed unmasking. However, due to the observed up-regulation of Xog1, it seems unlikely that it is the sole mechanism driving increased ß(1,3)-glucan exposure during hyperactive *STE11*^*ΔN467*^ expression, although it is possible that it still may be contributing to reduce unmasking to the levels reported in this publication.

### Cph1 induces a positive feedback loop and stimulates additional signaling pathways to mediate ß(1,3)-glucan unmasking

**Fig 9:**
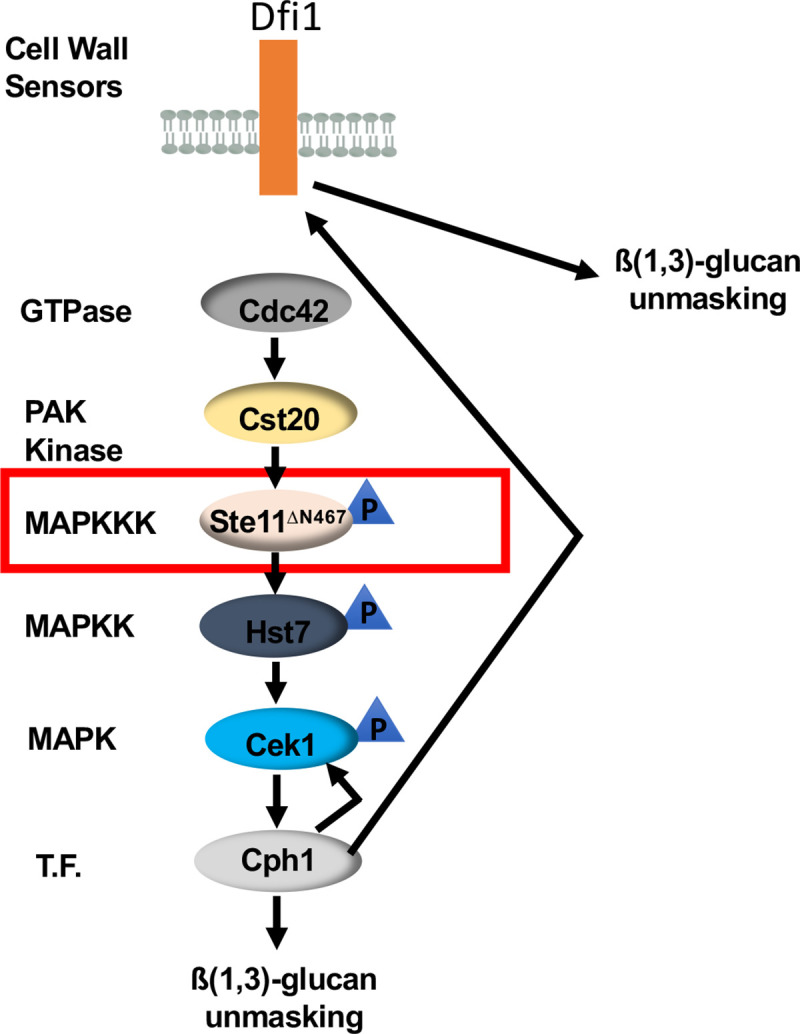
Proposed model for hyperactive *STE11*^*ΔN467*^ induced unmasking. In this model, expression of the hyperactive *STE11*^*ΔN467*^ allele (red box) induces activation of the canonical Cek1 MAPK pathway (Ste11-Hst7-Cek1) and activates the downstream transcription factor Cph1. Cph1 activation in turn induces increased ß(1,3)-glucan exposure through regulated expression of genes within its regulon, and mediates a positive feedback loop into the pathway to further hyperactivate the upstream MAPK Cek1 and increase its transcriptional output. Simultaneously, Cph1 activation causes an upregulation in the transcript levels of the cell wall sensor *DFI1*, which is then stimulated as a result of hyperactivation of the Cek1 MAPK pathway and initiates signaling in a parallel, unidentified, pathway that is also necessary to achieve the full levels of unmasking during *STE11*^*ΔN467*^ expression.

Previous RNA sequencing results have shown an up-regulation in the transcript levels of several components upstream of Cph1 in the Cek1 MAPK pathway, including *CEK1* and the cell wall sensor genes *DFI1* and *OPY2* [25]. Here, we have shown that in addition to inducing unmasking, Cph1 can regulate the activation of its own upstream MAPK, Cek1 (Fig 6). However, the total Cek1 levels were unchanged during *CPH1* deletion, suggesting that looping into the Cek1 MAPK pathway occurs at a different point than Cek1. Although the location in the pathway where the loop is occurring is thus far unknown, we originally hypothesized that this looping may take place at the cell wall sensor *DFI1*, a two transmembrane protein that has previously been implicated in Cek1 activation during invasive filamentation [48,49,59]. However, western blot analysis revealed that changes in *DFI1* expression levels did not alter Cek1 activation (Fig 8), suggesting that Dfi1 stimulates a parallel signaling pathway capable of regulating ß(1,3)-glucan exposure during *STE11*^*ΔN467*^ expression. We therefore propose a model in which active Cph1 functions in a positive feedback loop within the Cek1 MAPK pathway to mediate Cek1 activation, while simultaneously increasing the expression levels of the cell wall sensor Dfi1 to induce activation of a parallel, unidentified signaling pathway capable of regulating ß(1,3)-glucan exposure (Fig 9). Although this additional pathway has yet to be identified, Dfi1 has been found to have interactions with calmodulin [49], and may influence unmasking via this interaction. Alternatively, the *S*. *cerevisiae* cell wall sensor Mid2, a protein sharing strong structural similarity to Dfi1, has been shown to interact with the guanine nucleotide exchange factor Rom2, which is a regulator of Rho1 activation and the Mkc1 MAPK pathway [60]. We have previously shown that expression of a hyperactive *RHO1*^*Q67L*^ mutant is capable of inducing unmasking [26], and it may be possible that Dfi1 plays a similar role as Mid2 in stimulating this pathway during *STE11*^*ΔN467*^ expression.

Although it is unclear which signal cascade Dfi1 stimulates during hyperactive *STE11*^*ΔN467*^ expression, our data does suggest that the levels of *DFI1* expression are important for regulating the magnitude of the observed unmasking during *STE11*^*ΔN467*^ expression, as overexpression of *DFI1* on a third allele increased both unmasking (Fig 7C) and murine macrophage TNFα secretion *in vitro* (Fig 7F) for strains already expressing *STE11*^*ΔN467*^. This suggests that Dfi1 contributes to a graded response in signal transduction, where the strength of the response is dependent on the levels of Dfi1 in the cell. This is not uncommon for upstream components within a signal transduction pathway. In *Saccharomyces cerevisiae*, expression of the Gß subunit *STE4*, a component of the G-protein-coupled-receptor involved in α-pheromone sensing and mating, under the control of the inducible *GAL1* promoter showed a graded signaling response that was dependent on *STE4* expression levels to induce a corresponding increase in promoter activation of its downstream MAPK, Fus3 [61]. Thus, further research is warranted to identify how Dfi1 expression levels feed into appropriate signal cascades to mediate ß(1,3)-glucan exposure.

## Materials and methods

### Ethics statement

All animal work used in this study was done under an animal protocol that was previously approved by the University of Tennessee, Knoxville Institution Animal Care and Use Committee (IACUC), and was done in accordance with the National Institute of Health’s (NIH) ethical guidelines for animal research.

### Growth media and culture conditions

*C*. *albicans* strains were grown in YPD media (1% yeast extract, 2% peptone, 2% dextrose) at 30°C while shaking at 225 rpm [62]. To repress expression of the tetracycline repressible promoter, 0.5ug/ml of doxycycline (Sigma-Aldrich) was added to the growth media. Minimal media (0.67% yeast nitrogen base without amino acids, 2% dextrose, 2% agar) (Thermo Fisher Scientific) was used to select for loss of the genomically integrated guide RNA, Cas9, and nourseothricin resistance genes after CRISPR-Cas9 gene deletion [63]. YPM media (1% yeast extract, 2% peptone, 2% maltose)(Thermo Fisher Scientific) was used for flipping out the *SAT1*-flipper cassette [64]. DH5-α *Escherichia coli* strains (NEB) were cultured in LB media (0.5% yeast extract, 1% tryptone, 1% NaCl) (Thermo Fisher Scientific) and grown at 37°C on a rotator. RAW264.7 macrophages were cultured in Dulbecco’s modified eagle medium with L-glutamine (Gibco) containing 10% fetal bovine serum (Invitrogen) and 1% penicillin-streptomycin (Thermo Fisher Scientific) and grown at 37°C in 5% CO_2_ [65].

### Plasmid construction

Plasmids constructed during this project are listed in S1 Table and primers are listed in S2 Table. Unless otherwise stated all *C*. *albicans* genes and flanking regions were amplified from SC5314 *C*. *albicans* genomic DNA. The *LEU2* deletion construct was created using the *SAT1* flipper method [64]. The primer set AWO35 and AWO36 were used to amplify a 524bp fragment of the 5’ untranslated region (UTR) of *LEU2* that was flanked by *Kpn1* and *Xho1* cut sites introduced by the primers, respectively. The PCR fragment and the pSFS2A *SAT1* flipper cassette were then both digested with *KpnI* and *XhoI* and ligated together using T4 DNA ligase (NEB) to create the pAW006 plasmid. To introduce the 3’ UTR of *LEU2* to the 3’ end of pAW006, the primer set AWO37 and AWO38 were used to amplify a 520bp fragment of the 3’ UTR of *LEU2* that was flanked by *SacII* and *SacI* cut sites introduced by each primer, respectively. Both the PCR product and pAW006 were then digested with *SacII* and *SacI*, and ligated together to create the pAW011 plasmid that harbors the full *LEU2-SAT1* flipper cassette.

The *CPH1-FLAG* reintegrate cassette was created using the *SAT1* flipper cassette pSFS2A [64]. A 484bp fragment of the 3’ UTR of *CPH1* was amplified using TC0173 and TCO174 that additionally introduced *NotI* and *SacI* cut sites, respectively, on the flanking ends. Both pSDS2A and the PCR fragment were digested with *NotI* and *SacI* restriction enzymes (NEB) and ligated using T4 DNA ligase (NEB) to create pTCO74. To introduce a tagged *CPH1* gene to the 5’ end of the *SAT1* flipper cassette in the pTCO74 plasmid, a 2,361bp fragment containing 326bp of the *CPH1* 5’ UTR immediately upstream of the start codon and the *CPH1* open reading frame (ORF) with an added C-terminal FLAG-tag sequence before the stop codon was amplified using AWO187 and AWO188, which further introduced flanking *ApaI* and *XhoI* cut sites, respectively. The final *CPH1-Flag-SAT1* reintegrate cassette (pAW071) was then created by digesting both pTCO74 and the PCR product with *ApaI* and *XhoI*, and subsequently ligating the digested vector and insert together.

The *CEK1* overexpression cassette was created by placing *CEK1* under the control of the constitutive phosphoenolpyruvate carboxykinase (*PCK1*) promoter in the *SAT1*-flipper-cassette pSFS2A background. To create this, a PCR fragment containing 495bp of the 3’ UTR of *PCK1* was amplified with AWO265 and AWO266 primers that introduced *SacII* and *SacI* cut sites, respectively. The PCR product and pSFS2A were then digested using *SacII* and *SacI* and ligated to create pAW085. To introduce the *PCK1* promoter to the 5’ end of the *SAT1* flipper cassette in pAW085, a PCR product consisting of the 712bp in the 5’ UTR of *PCK1* that sits immediately upstream of the *PCK1* ORF was amplified with the AWO263 and AWO264 primer set that introduced flanking *KpnI* and *ApaI* cut sites, respectively. The plasmid and 5’ UTR PCR product were then subsequently digested with *KpnI* and *ApaI* and ligated to create the *P*_*PCK1*_*-SAT1* flipper cassette (pAW087). *CEK1* was then subcloned under the control of the *PCK1* promoter by amplifying the *CEK1* ORF with the AWO267 and AWO268 primer set that introduced flanking *ApaI* and *XhoI* cut sites, respectively. pAW087 and the *CEK1* ORF PCR product were then digested with *ApaI* and *XhoI*, and subsequently ligated to create the *P*_*PCK1*_*-CEK1-SAT1* flipper cassette (pAW091).

The *HST7* overexpression cassette was created by placing the *HST7* gene under the control the phosphoenolpyruvate carboxykinase (*PCK1*) promoter in plasmid pAW087. The *HST7* ORF was amplified with the primer set AWO348 and AWO349 with flanking *ApaI* cut sites flanking the ends. pAWO87 and the *HST7* ORF PCR product were then digested with *ApaI* and ligated together to create the *P*_*PCK1*_*-HST7-SAT1* flipper cassette (pAW105).

The *DFI1* overexpression cassette was created by placing the *DFI1* ORF under the regulatory control of the constitutive enolase *(ENO1*) promoter [66]. To achieve this, a 1,529bp PCR product consisting of the *DFI1* ORF and 441bp of the *DFI1* 3’ UTR immediately following the stop codon was amplified with the AWO176 and AWO177 primer set that introduced flanking *NotI* and *SacI* cut sites. The *P*_*ENO1*_ overexpression plasmid, pBT1 [66], and the PCR product were then digested using *NotI* and *SacI*, and ligated to create the *P*_*ENO1*_*-DFI1-SAT1* overexpression cassette (pAW066).

### Strain construction

*Candida albicans* deletion strains (S3 Table) were created with the use of CRISPR-Cas9 as previously described [63]. The *LEU2/leu2Δ* mutant was generated with the use of the *SAT1* flipper method [64]. The *LEU2* deletion construct (pAW011) was digested with *KpnI* and *SacI*, and the fragment containing the *SAT1* gene flanked by homologous regions to the 5’ and 3’ UTRs of *LEU2* was gel purified with the use of the QIAquick Gel Extraction Kit (Qiagen). The purified fragment was then transformed via electroporation into the SC5314 *C*. *albicans* strain as previously described [54], and successful transformants were selected on YPD plates containing 200μg/ml nourseothricin.

To generate the *STE11/P*_*tet-off*_*-STE11*^*ΔN467*^ strain in the SC5314 *C*. *albicans* background, the primer set AWO156 and AWO157 were used to amplify the tetracycline repressible promotor with an additional ~500bp of homology flanking both sides of the *STE11* locus (*P*_*tet-off*_*-STE11*^*ΔN467*^) from purified genomic DNA of the previously constructed *STE11/P*_*tet-off*_*-STE11*^*ΔN467*^ strain in the Day286 background [25]. The resulting PCR product contained the Hygromycin selectable marker and the tetracycline repressible promotor 5’ to the *P*_*tet-off*_ -regulated N-terminally truncated *STE11*^*ΔN467*^ allele. The PCR product was then purified and transformed into the appropriate strains via electroporation and transformants were selected for on YPD+HYG plates containing 2mg/ml of Hygromycin B (Gold Biotechnology).

The *CPH1-FLAG* reintegrate strain was generated with the use of the *SAT1-*flipper method [64]. The *CPH1-FLAG-SAT1* reintegrate cassette (pAW071) was digested using *ApaI* and *SacI*, and subsequently purified using the QIAquick Gel Extraction Kit (Qiagen). The purified fragment was then transformed into the appropriate strains via electroporation and transformants were selected on YPD + nourseothricin plates.

The *CEK1* and *HST7* overexpression mutants were generated by digestion of the *P*_*PCK1*_*-CEK1-SAT1* flipper cassette (pAW091) or the *P*_*PCK1*_*-HST7-SAT1* flipper cassette (pAW105) with *KpnI*, followed by purification of the linear plasmid with the QIAquick PCR Purification Kit (Qiagen). The linear plasmid was then transformed into the appropriate *C*. *albicans* strains via electroporation and transformants selected for on YPD + nourseothricin media.

*DFI1* overexpression strains were created by digesting the *P*_*ENO1*_*-DFI1-SAT1* plasmid (pAW066) with *MscI* (which cuts a single time within the *ENO1* promoter). The linear plasmid was then purified and transformed into the appropriate *C*. *albicans* strains via electroporation. Successful transformants were selected for on YPD + nourseothricin media.

### Mouse model

Outbred ICR mice (ENVIGO) were used for all experiments in this study. To prepare for inoculation, 50ml cultures of *C*. *albicans* strains were grown overnight in YPD media containing 0.5μg/ml of doxycycline (Sigma-Aldrich) at 30°C while shaking at 225 rpm. The following morning, cells were transferred to 50ml conical tubes (Thomas Scientific) and spun at 3,500 rpm for 5 minutes. Cells were subsequently washed 2 times with 25ml of PBS and then counted using a hemocytometer. Cells were diluted to either 1x10^7^ cells/ml (for immunocompetent mice) or 1x10^5^ cells/ml (for immunosuppressed mice). Mice were then intravenously injected via the lateral tail vein with 0.1ml of the previously prepared cell suspension. Following injection, cell viability for each strain was assessed by plating on YPD and allowing cells to grow at 30°C for 24 hours. Four days post infection, mice were anesthetized, and peripheral blood was isolated via cardiac puncture in heparin treated tubes (BD Biosciences). Mice were then euthanized, and their kidneys, spleens, brains or livers were harvested to assess fungal burden. Organs were placed in pre-weighed whirl-pack bags (Thermo Fisher Scientific) containing 1ml of water. The bags were weighed once more to determine the weight of the organ and then the tissues were homogenized. Serial dilutions of the tissue homogenates (10^−1^, 10^−2^, 10^−3^) were created, and 1ml of each dilution was added to 15ml of YPD + 75μg/ml chloramphenicol (Thermo Fisher Scientific) precooled to 55°C and plated. Plates were then left to incubate for 2 days at 30°C to determine fungal colony forming units (CFU) per gram of tissue. Distribution normality of the data collected was then assessed with the use of a Shapiro-Wilk test and statistical significance was determined with the use of either Student’s unpaired T-test for data that followed a Gaussian distribution, or a non-parametric Kruskal-Wallis test for data that did not follow a Gaussian distribution (GraphPad Prism, v7.0c software) (The number of mice used for each experiment is listed in the figure legend associated with the data).

For experiments in which the regulation of the hyperactive *STE11*^*ΔN467*^ allele was controlled *in vivo*, mice were given either 5% sucrose water or 5% sucrose + 2mg/ml of doxycycline [25]. Three days prior to infection, all mice were given sucrose water to begin acclimation. Two days prior to infection, mice in the appropriate control groups were then switched to sucrose water + doxycycline. All supplied water was then changed every other day to ensure the efficacy of the supplemented doxycycline.

### Immune cell depletion in mice

Non-specific immune cell depletion *in vivo* was achieved via recurring injections with cyclophosphamide (Sigma-Aldrich). ICR mice were weighed prior to treatment to determine the average weight of all mice. Mice were then treated with 150mg/kg of cyclophosphamide, based on their average weight, via intraperitoneal (I.P.) injections starting 4 days prior to infection. Immune depletion was then maintained with recurring I.P. injections of 150mg/kg of cyclophosphamide every 3 days until the experiment was terminated.

*In vivo* neutrophil depletion was achieved through the use of an anti-mouse Ly6G (1A8) monoclonal antibody (BioXCell) [29,30]. Mice were intraperitoneally injected with 300ug of 1A8 starting 1 day prior to infection. Neutrophil depletion was then maintained via recurring I.P. administration of 300ug of 1A8 every other day until the end of the experiment. Control mice were alternatively injected with a corresponding I.P. injection with a PBS vehicle control.

### Staining of cell wall components and flow cytometry or microscopy analysis

For staining of yeast cells, 5ml cultures of *C*. *albicans* strains in YPD were started the morning prior to staining. Cultures were left to grow shaking at 225 rpm at 30°C for ~8 hours, and then back diluted to an OD_600_ of 0.1 in fresh YPD media. Dilutions were then incubated with shaking at 225 rpm and 30°C overnight (~16 hours) for staining the following morning. After overnight growth, cells were diluted to an OD_600_ of 0.5. Immunofluorescent staining of exposed ß(1,3)-glucan in stationary phase yeast cells was performed as previously described [26]. Total chitin was assessed by staining cells with 500μl of a 10μg/ml calcofluor white solution in PBS for 5 minutes shaking at room temperature. To assess exposed chitin, cells were stained with 500μl of a 10ug/ml fluorescein-labeled wheat germ agglutinin (WGA)(Vector Laboratories) solution in PBS and incubated with shaking at 4°C for 30 minutes. Mannan levels were assessed by staining cells with 500μl of a 50ug/ml solution of Alexa Fluor 647 conjugated concanavalin A (Thermo Fisher Scientific) in PBS and were incubated with shaking at 4°C for 30 minutes. A minimum of 3 biological replicates with 2–3 technical replicates consisting of 100,000 recorded events each was used to assess all strains by flow cytometry and statistical significance was determined with the use of a one-way ANOVA with Tukey’s post hoc analysis (GraphPad Prism, v7.0c software).

Immunofluorescent staining of hyphal cells for exposed ß(1,3)-glucan was performed as previously described [51]. Quantification of microscopy images was then achieved with the use of ImageJ (National Institute of Health, Bethesda, MD). A total of 3 biological replicates were stained for each strain and 30 hyphal cells were quantified per biological replicate. Statistical significance was then determined with the use of Student’s t-test (GraphPad Prism, v7.0c software).

Immunofluorescent staining of leukocytes was achieved using peripheral blood that was isolated via cardiac puncture in heparin treated tubes (BD Biosciences). Blood was resuspended in 1x PBS and erythrocytes were lysed using two sequential ACK (Ammonium-Chloride-Potassium) lysis steps. Cells were then counted and stained using myeloid markers (α-Ly6C: HK1.4 clone, APC, BioLegend) (α-Ly6G: 1A8 clone, PerCP-Cy5.5, BioLegend) (α-CD11b: M1/70 clone, BV421, BioLegend) (α-F4/80: BM8 clone, PE, BioLegend) (Fixable NIR Live-Dead: under APC-Cy7 filter, Invitrogen; cat #:L34975).

For all stains, relative abundance and representative plots were generated using an LSRII flow cytometer (BD Biosciences), and analysis was performed using FlowJo v10.7 (Becton, Dickinson and Company). For strains in which microscopy was performed, cells were analyzed using the Leica SP8 confocal microscope and analyzed using the LAS X analysis software (Leica).

### Serum cytokine quantification

Serum cytokine concentrations were determined using the manufacturer’s protocol for the LEGENDplex Mouse Anti-Virus Response Panel 740622 (Biolegend). In brief, peripheral blood from anesthetized mice was collected and allowed to clot for 30 minutes prior to centrifugation at 1,000 x g for 20 minutes for serum separation. Serum was then collected and stored at -80°C until use. Frozen samples were then allowed to thaw completely and diluted 2-fold for kit analysis. All assays were performed using the manufacturer supplied 96-well v-bottom plate. Once samples were thawed and diluted, each well was filled with 25μl of the appropriate sample or standard, 25μl of assay buffer, and 25μl of capture beads. The plate was then covered and left shaking at 800 rpm at room temperature for 2 hours. Following incubation, beads were pelleted by centrifugation at 200 x g for 5 minutes, the supernatant was removed, and beads were washed two times with 200μl of 1X wash buffer. Detection antibody (25μl) was then added to each well, and the plate was left to incubate covered at room temperature for 1 hour while shaking at 800 rpm. Following incubation, 25μl of SA-PE was added to each well and plates were left to incubate shaking in the dark for 30 minutes. Samples were then spun at 200 x g for 5 minutes, washed one time with 200μl of 1X wash buffer and finally resuspended in 150μl of wash buffer. Following resuspension, samples were analyzed via flow cytometry using an LSRII flow cytometer (BD Biosciences), and data was analyzed using the manufacturer supplied LEGENDplex data analysis software suite (Biolegend). Distribution normality of the data collected was then assessed with the use of a Shapiro-Wilk test and statistical significance was determined by a Mann-Whitney test. (The number of mice used for each experiment is listed in the figure legend associated with the data and all samples were run with technical duplicates).

### TNFα Enzyme Linked Immunosorbent Assay (ELISA)

One day prior to infection, 5x10^5^ RAW264.7 murine macrophages were seeded in each well of a 24-well plate and left to incubate overnight at 37°C in 5% CO_2_. Cultures of *C*. *albicans* strains in YPD were then started and left to grow shaking at 225 rpm at 30°C overnight. The following morning, *C*. *albicans* cultures were washed with PBS and diluted to an OD_600_ of 1.25 in 5ml of PBS in a 6-well plate. Diluted cultures were then UV-inactivated by placing the 6-well plate in the Spectrolinker XL-1000 UV Crosslinker (Spectroline Inc.) and adjusting the ENERGY setting to 100,000 μJ/cm^2^. UV-irradiation was then repeated for a total of 5 exposures. UV-inactivated *Candida* cells, or a PBS control, were then added to the RAW264.7 macrophages at 1:10 macrophage to *Candida* ratio and left to co-incubate for 4 hours at 37°C in 5% CO_2_. For analysis of the impact that dectin-1 binding had on TNFα production, macrophages were co-incubated with 5ug/ml of an anti-dectin-1 neutralizing antibody (InvivoGen) for 1 hour prior to co-incubation with UV-inactivated *C*. *albicans* cells. After *C*. *albicans* and macrophage co-incubation, the supernatant was collected and filtered through a 0.22μm filter (Thermo Fisher Scientific). Filtered supernatant was then assessed with the use of a Duoset Mouse TNFα ELISA kit (R&D Systems) per the manufacturer’s instructions. Three biological replicates with three technical replicates were used to assess each strain, and statistical significance was determined with the use of a one-way ANOVA with Tukey’s post hoc analysis (GraphPad Prism, v7.0c software).

### Western blot analysis of total and phosphorylated-Cek1

Total and activated levels of Cek1 were assessed as previously described [25]. After blotting, the total levels of phosphorylated-Cek1 to total Cek1 was determined via densitometry of images generated on the Odyssey Imager (Li-Cor Biosciences) by using ImageJ (National Institute of Health, Bethesda, MD). Quantification was done for three biological replicates of each sample, and statistical significance was determined with the use of a one-way ANOVA with Tukey’s post hoc analysis (GraphPad Prism, v7.0c software).

### Statistics

All statistical analyses used are stated in the methods section corresponding to the assay performed. For all figures, statistical significance between two samples is denoted by a horizontal line, where the end points of the line indicate the two samples that were compared to generate significance.

## Supporting information

S1 Fig Characterization of serum cytokines during systemic infection with a hyperactive *STE11^ΔN467^* mutant. ICR mice were intravenously infected with 1x10^6^ cells of *C*. *albicans* wild-type (Day286) or the *STE11/P*_*tet-off*_*-STE11*^*ΔN467*^ strain. Serum was collected 4 days post infection and the concentrations of CCL2, IL-1ß, IL-12, GM-CSF, CXCL1, IL-10, IFN-ß and IFN-γ were determined via flow cytometry using the LEGENDplex cytokine bead based array kit. (n = 8 mice)(*p<0.05, by Mann-Whitney test)(Horizontal dotted lines indicate the kit detection limit for each cytokine).
(TIF)

S2 Fig Representative Staining of Peripheral Blood during immunosuppression by cyclophosphamide. Peripheral blood samples from Cyclophosphamide (Cyclo) and PBS treated mice were analyzed for circulating myeloid cells. Mice were anesthetized, and peripheral blood was isolated via cardiac puncture in heparin treated tubes (BD Biosciences; San Jose, CA). Erythrocytes were lysed using 2 ACK lysis steps (ref), cells were counted and stained using myeloid markers (α-Ly6C: HK1.4, APC, BioLegend) (α-Ly6G: 1A8, PerCP-Cy5.5, BioLegend) (α-CD11b: M1/70 clone, BV421, BioLegend) (α-F4/80: BM8, PE, BioLegend) (Fixable NIR Live-Dead: APC-Cy7, Invitrogen; cat #:L34975). Relative abundance and representative plots were generated using an LSRII flow cytometer (BD Biosciences; San Jose, CA), and analysis was performed using FlowJo (Becton, Dickinson and Company; Ashland, OR). Flow cytometric analysis shows a marked decrease in circulating Monocytes (Lyc6C+/ CD11b+) and Neutrophils (Ly6G+/ CD11b+) after three doses of Cyclo (B) versus PBS (A).
(TIF)

S3 Fig Representative scatter plots of leukocyte populations during 1A8 neutrophil depletion. Peripheral blood of 1A8 depleted mice and mice receiving the PBS depletion control were analyzed for circulating myeloid cells. Myeloid populations were assessed via staining using myeloid markers (α-Ly6C: HK1.4, APC, BioLegend) (α-Ly6G: 1A8, PerCP-Cy5.5, BioLegend) (α-CD11b: M1/70 clone, BV421, BioLegend) (α-F4/80: BM8, PE, BioLegend) (Fixable NIR Live-Dead: APC-Cy7, Invitrogen; cat #:L34975). Relative abundance and representative plots were generated using an LSRII flow cytometer (BD Biosciences), and analysis was performed using FlowJo (Becton, Dickinson and Company). Flow cytometric analysis revealed that the levels of Ly6C+/Ly6G+ cells were markedly decreased in (A) 1A8 treated mice, when compared to (B) PBS treated mice at day 0 following infection with Day286 wild-type *C*. *albicans* cells. (C) 1A8 treated mice, when compared to (D) PBS treated mice at day 4 post infection following infection with Day286 wild-type *C*. *albicans* cells.
(TIF)

S4 Fig Kidney fungal burden in 1A8 control mice treated with PBS and infected with 1x10^4^ cells of the *STE11/Ptet-off-STE11^ΔN467^* or wild-type strains. Starting one day prior to infection (day -1), ICR mice were treated every 3 days with recurring injections of 200μl of PBS intraperitoneally as a drug vehicle control for 1A8 treatment. At day 0, mice were then intravenously infected with 1x10^4^ cells of *C*. *albicans* wild-type (Day286) or the *STE11/P*_*tet-off*_*-STE11*^*ΔN467*^ strain and their kidneys were harvested at 4 days post infection (d.p.i.) to assess fungal burden. (n = 5 mice)
(TIF)

S5 Fig Gating strategy for yeast cell population of *C. albicans*. (TIF)

S6 Fig *CEK1* deletion in the CAI4 wild-type background does not induce unmasking. Overnight cultures of wild-type and *cek1ΔΔ* mutant cells in the CAI4 background were stained with anti-ß(1,3)-glucan antibody and a phycoerythrin-conjugated secondary antibody for flow cytometry analysis to assess the levels of ß(1,3)-glucan exposure. Three biological replicates were run for each sample. (p = 0.2828, by student’s t-test)
(TIF)

S7 Fig The *hst7ΔΔ* mutation blocks ß(1,3)-glucan unmasking by hyperactive *STE11^ΔN467^*. Overnight cultures of the wild-type and *hst7ΔΔ* mutants were stained with an anti-ß(1,3)-glucan antibody and a phycoerythrin-conjugated secondary antibody followed by flow cytometry analysis to assess the levels of ß(1,3)-glucan exposure (****p<0.0001, by one-way ANOVA with Tukey’s post hoc analysis).
(TIF)

S1 Table Plasmids used in this study. (DOCX)

S2 Table Primers used in this study. (XLSX)

S3 Table *C. albicans* strains used in this study. (DOCX)
