# Supplementary figures and images for: Activation of Cph1 causes ß(1,3)-glucan unmasking in Candida albicans and attenuates virulence in mice in a neutrophil-dependent manner
Source: PLoS Pathog. 2021 Aug 25;17(8):e1009839. doi: 10.1371/journal.ppat.1009839 (PMC8423308; doi:10.1371/journal.ppat.1009839)

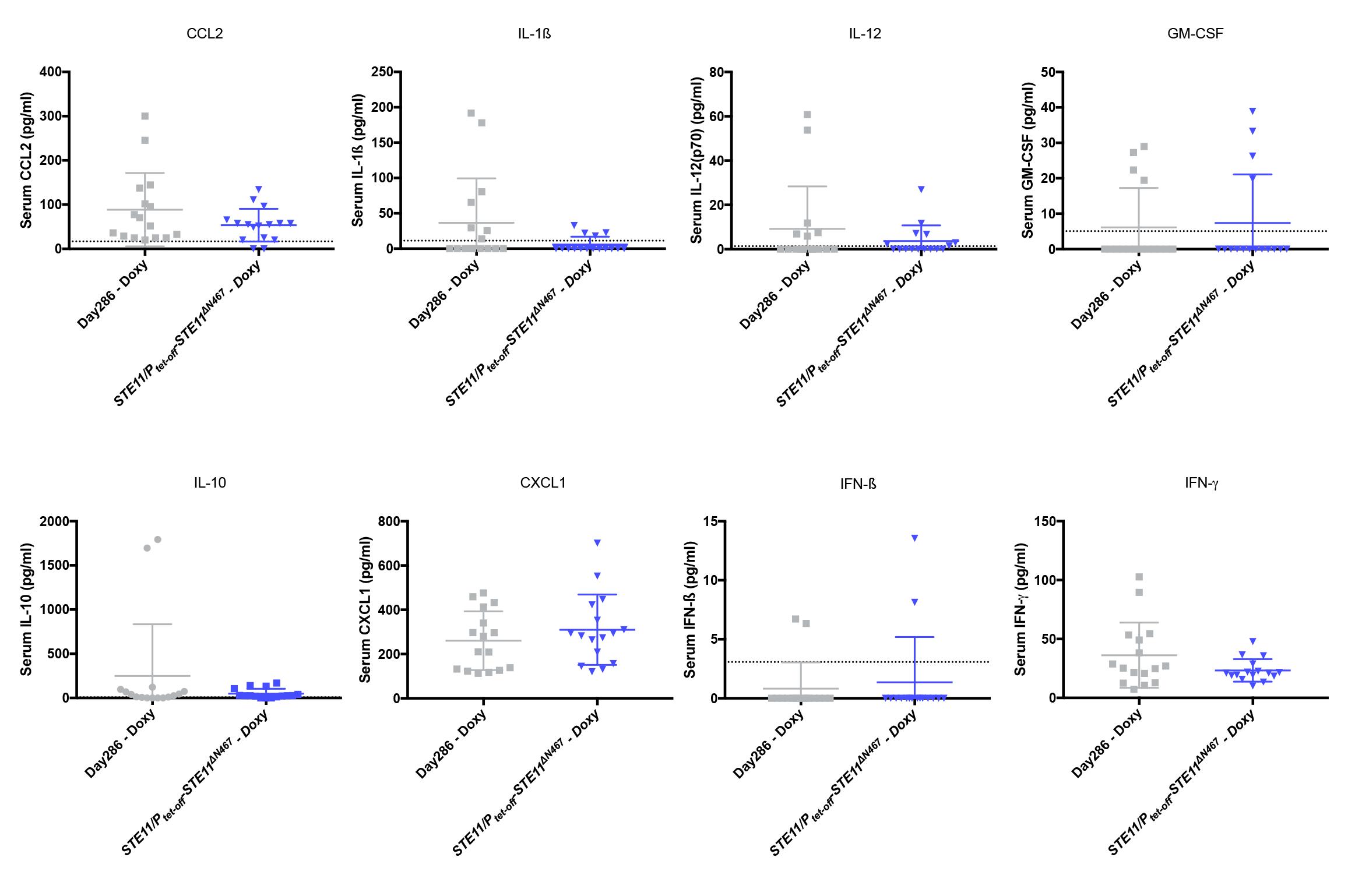

Supplement: S1 Fig — ICR mice were intravenously infected with 1x10^6^ cells of C. albicans wild-type (Day286) or the STE11/Ptet-off-STE11ΔN467 strain. Serum was collected 4 days post infection and the concentrations of CCL2, IL-1ß, IL-12, GM-CSF, CXCL1, IL-10, IFN-ß and IFN-γ were determined via flow cytometry using the LEGENDplex cytokine bead based array kit. (n = 8 mice)(*p<0.05, by Mann-Whitney test)(Horizontal dotted lines indicate the kit detection limit for each cytokine). (TIF) [file ppat.1009839.s001.tif]

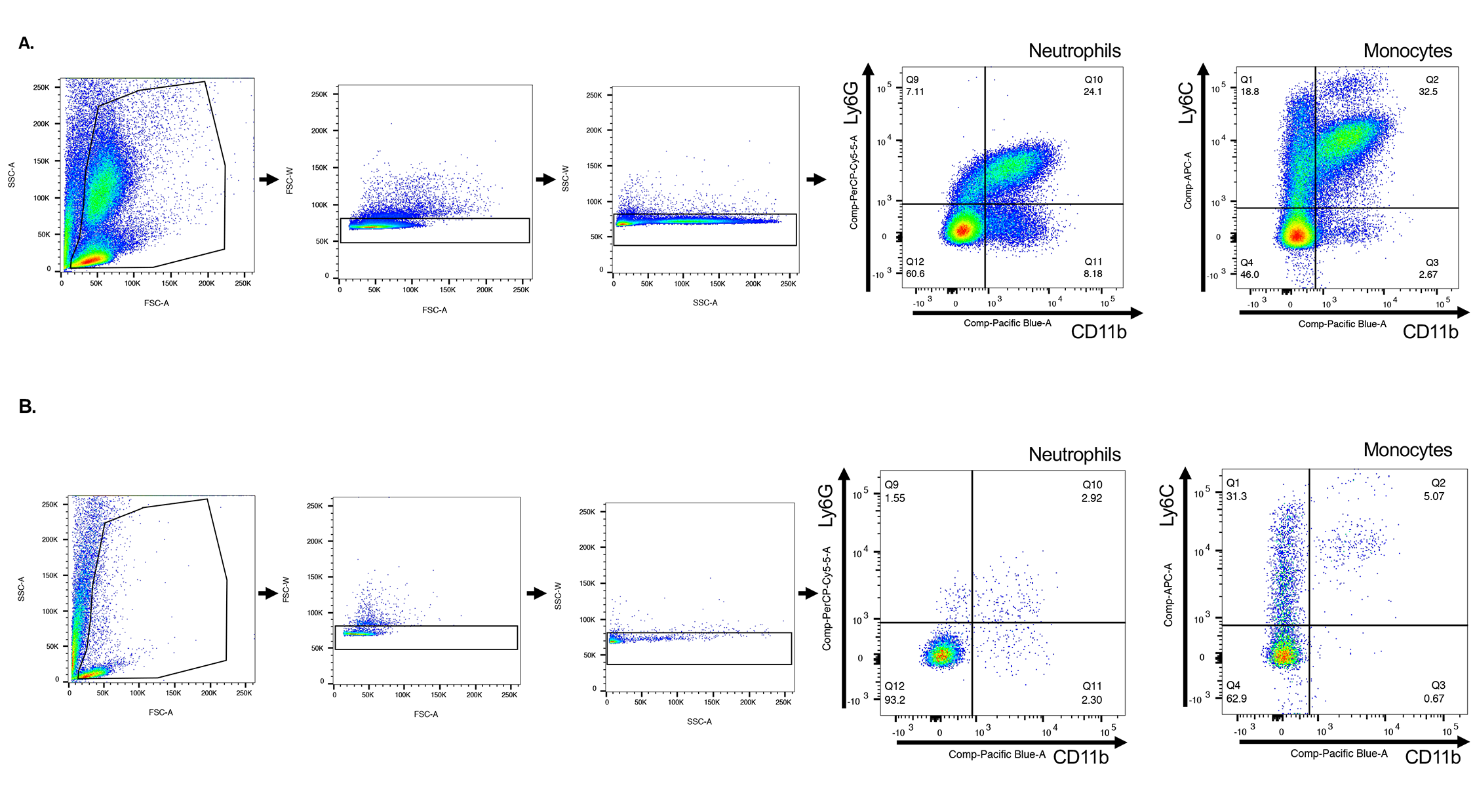

Supplement: S2 Fig — Peripheral blood samples from Cyclophosphamide (Cyclo) and PBS treated mice were analyzed for circulating myeloid cells. Mice were anesthetized, and peripheral blood was isolated via cardiac puncture in heparin treated tubes (BD Biosciences; San Jose, CA). Erythrocytes were lysed using 2 ACK lysis steps (ref), cells were counted and stained using myeloid markers (α-Ly6C: HK1.4, APC, BioLegend) (α-Ly6G: 1A8, PerCP-Cy5.5, BioLegend) (α-CD11b: M1/70 clone, BV421, BioLegend) (α-F4/80: BM8, PE, BioLegend) (Fixable NIR Live-Dead: APC-Cy7, Invitrogen; cat #:L34975). Relative abundance and representative plots were generated using an LSRII flow cytometer (BD Biosciences; San Jose, CA), and analysis was performed using FlowJo (Becton, Dickinson and Company; Ashland, OR). Flow cytometric analysis shows a marked decrease in circulating Monocytes (Lyc6C+/ CD11b+) and Neutrophils (Ly6G+/ CD11b+) after three doses of Cyclo (B) versus PBS (A). (TIF) [file ppat.1009839.s002.tif]

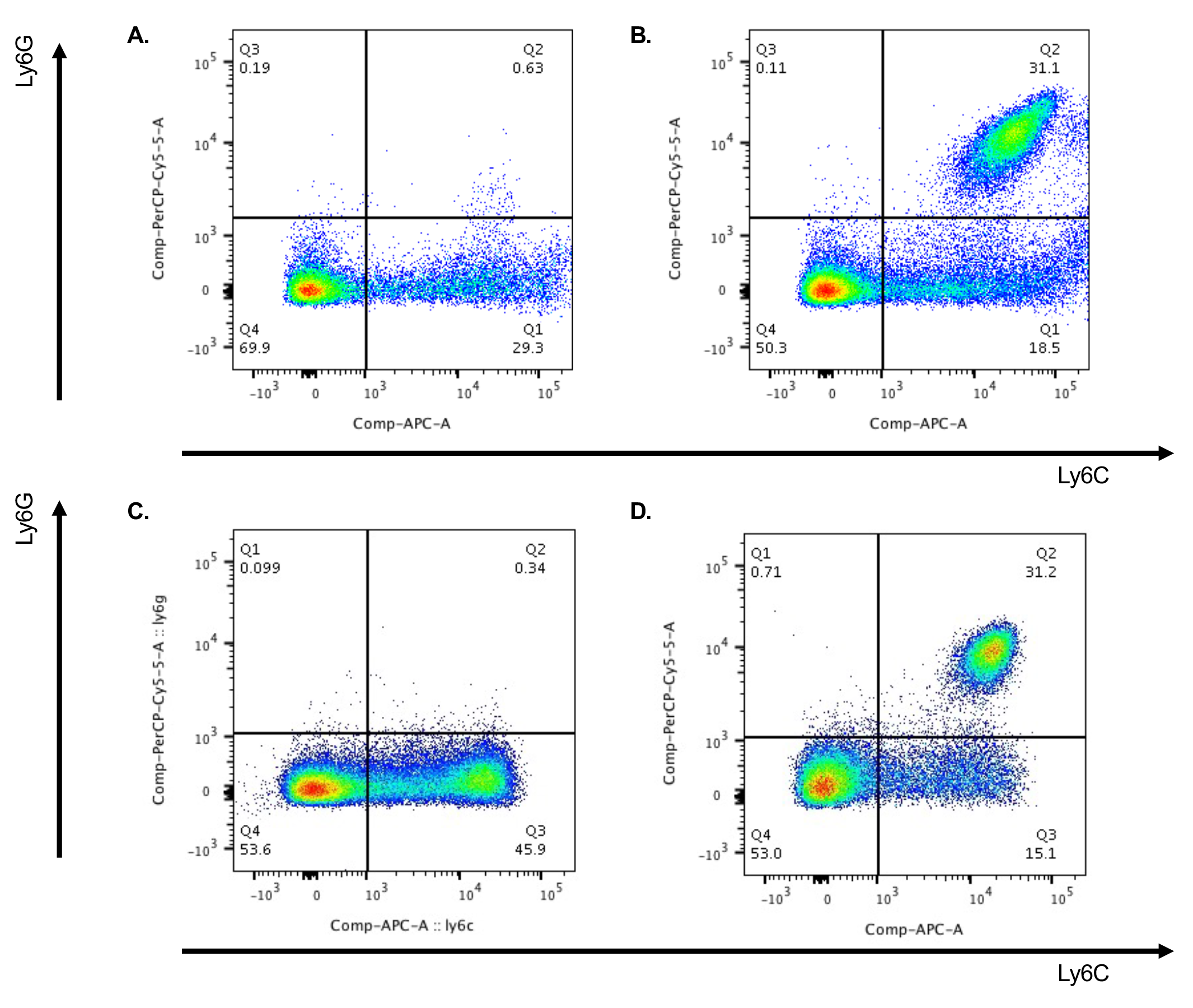

Supplement: S3 Fig — Peripheral blood of 1A8 depleted mice and mice receiving the PBS depletion control were analyzed for circulating myeloid cells. Myeloid populations were assessed via staining using myeloid markers (α-Ly6C: HK1.4, APC, BioLegend) (α-Ly6G: 1A8, PerCP-Cy5.5, BioLegend) (α-CD11b: M1/70 clone, BV421, BioLegend) (α-F4/80: BM8, PE, BioLegend) (Fixable NIR Live-Dead: APC-Cy7, Invitrogen; cat #:L34975). Relative abundance and representative plots were generated using an LSRII flow cytometer (BD Biosciences), and analysis was performed using FlowJo (Becton, Dickinson and Company). Flow cytometric analysis revealed that the levels of Ly6C+/Ly6G+ cells were markedly decreased in (A) 1A8 treated mice, when compared to (B) PBS treated mice at day 0 following infection with Day286 wild-type C. albicans cells. (C) 1A8 treated mice, when compared to (D) PBS treated mice at day 4 post infection following infection with Day286 wild-type C. albicans cells. (TIF) [file ppat.1009839.s003.tif]

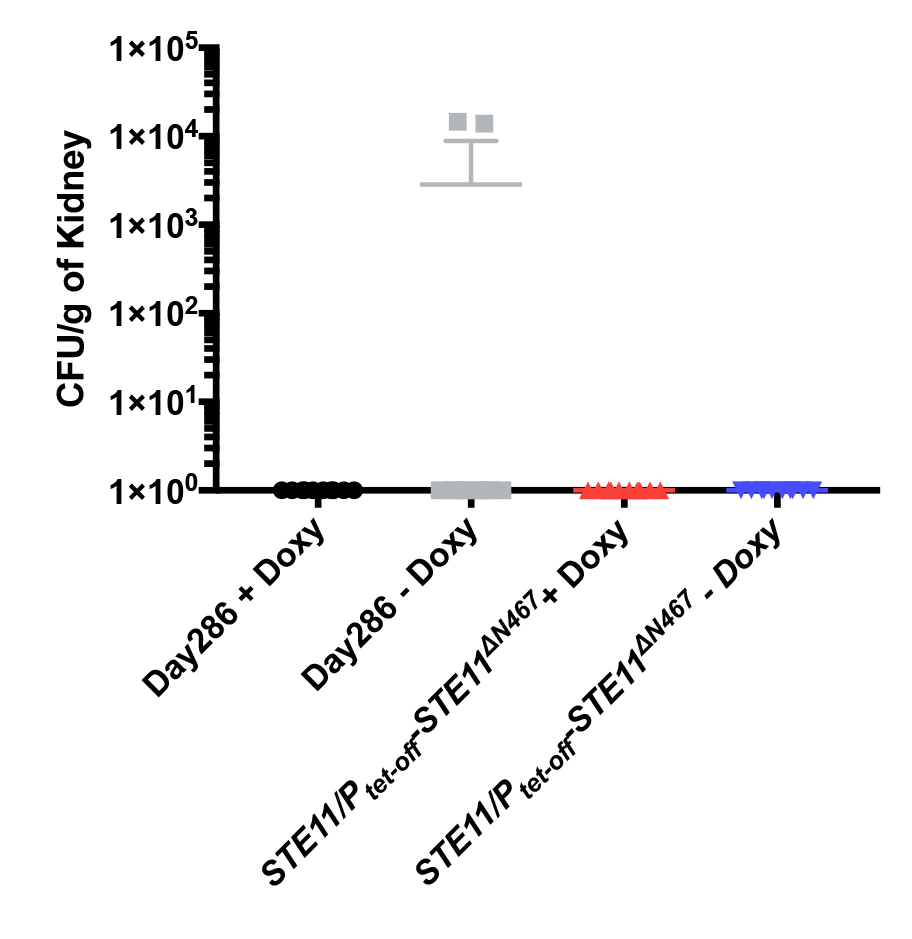

Supplement: S4 Fig — Starting one day prior to infection (day -1), ICR mice were treated every 3 days with recurring injections of 200μl of PBS intraperitoneally as a drug vehicle control for 1A8 treatment. At day 0, mice were then intravenously infected with 1x10^4^ cells of C. albicans wild-type (Day286) or the STE11/Ptet-off-STE11ΔN467 strain and their kidneys were harvested at 4 days post infection (d.p.i.) to assess fungal burden. (n = 5 mice) (TIF) [file ppat.1009839.s004.tif]

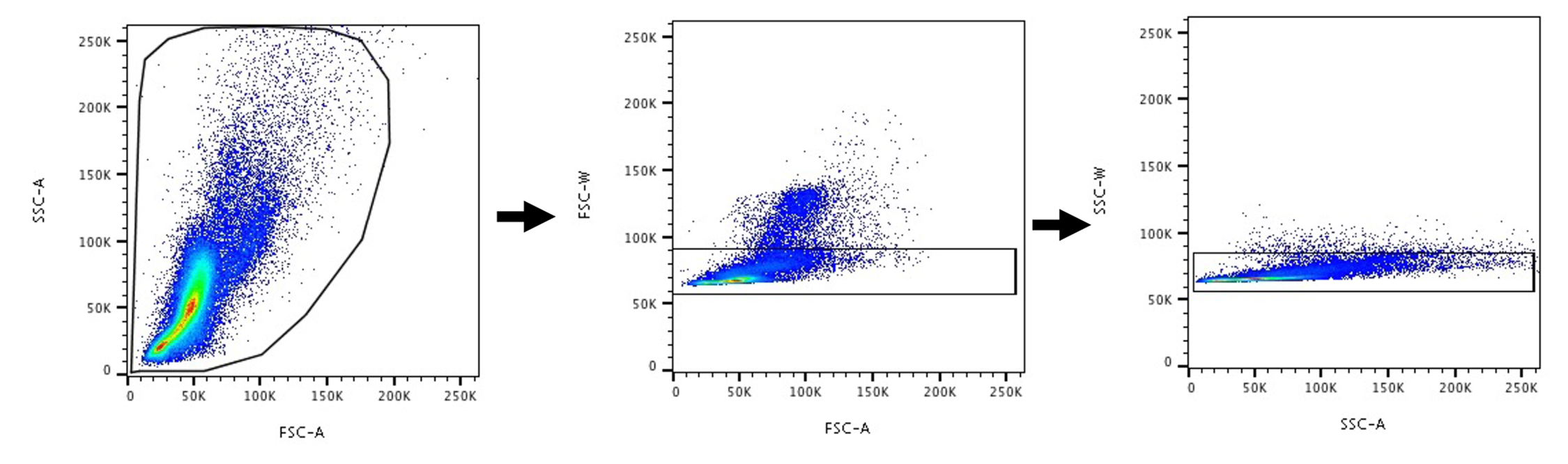

Supplement: S5 Fig — (TIF) [file ppat.1009839.s005.tif]

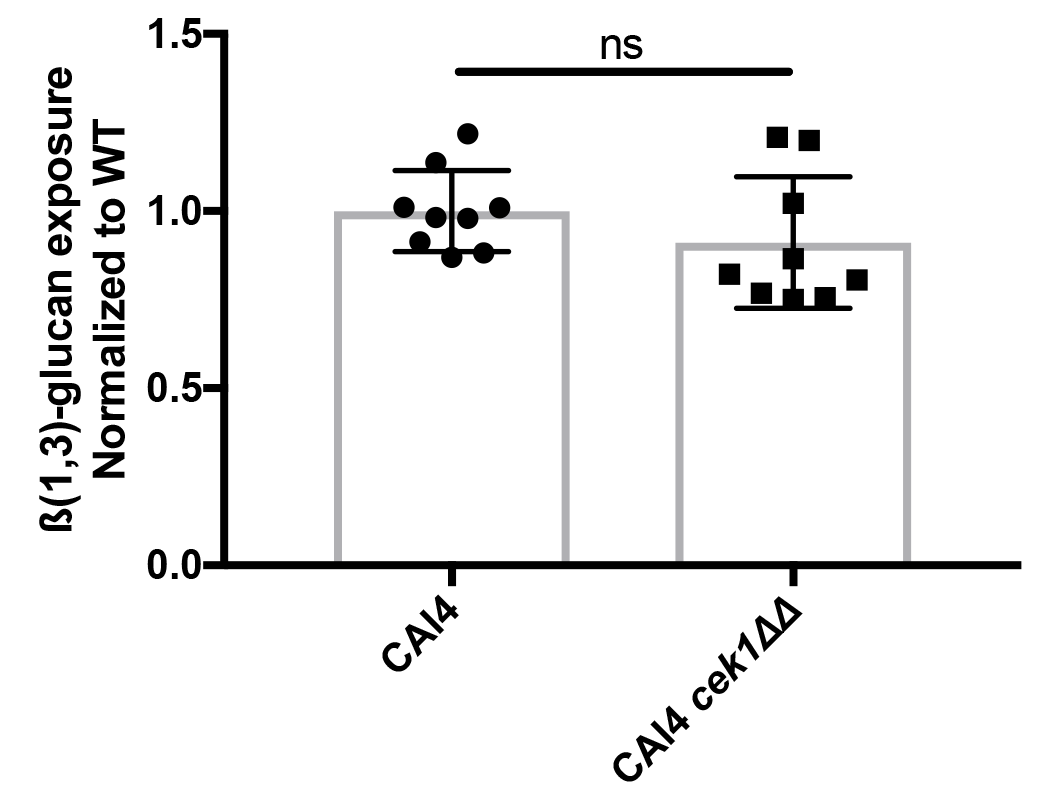

Supplement: S6 Fig — Overnight cultures of wild-type and cek1ΔΔ mutant cells in the CAI4 background were stained with anti-ß(1,3)-glucan antibody and a phycoerythrin-conjugated secondary antibody for flow cytometry analysis to assess the levels of ß(1,3)-glucan exposure. Three biological replicates were run for each sample. (p = 0.2828, by student’s t-test) (TIF) [file ppat.1009839.s006.tif]

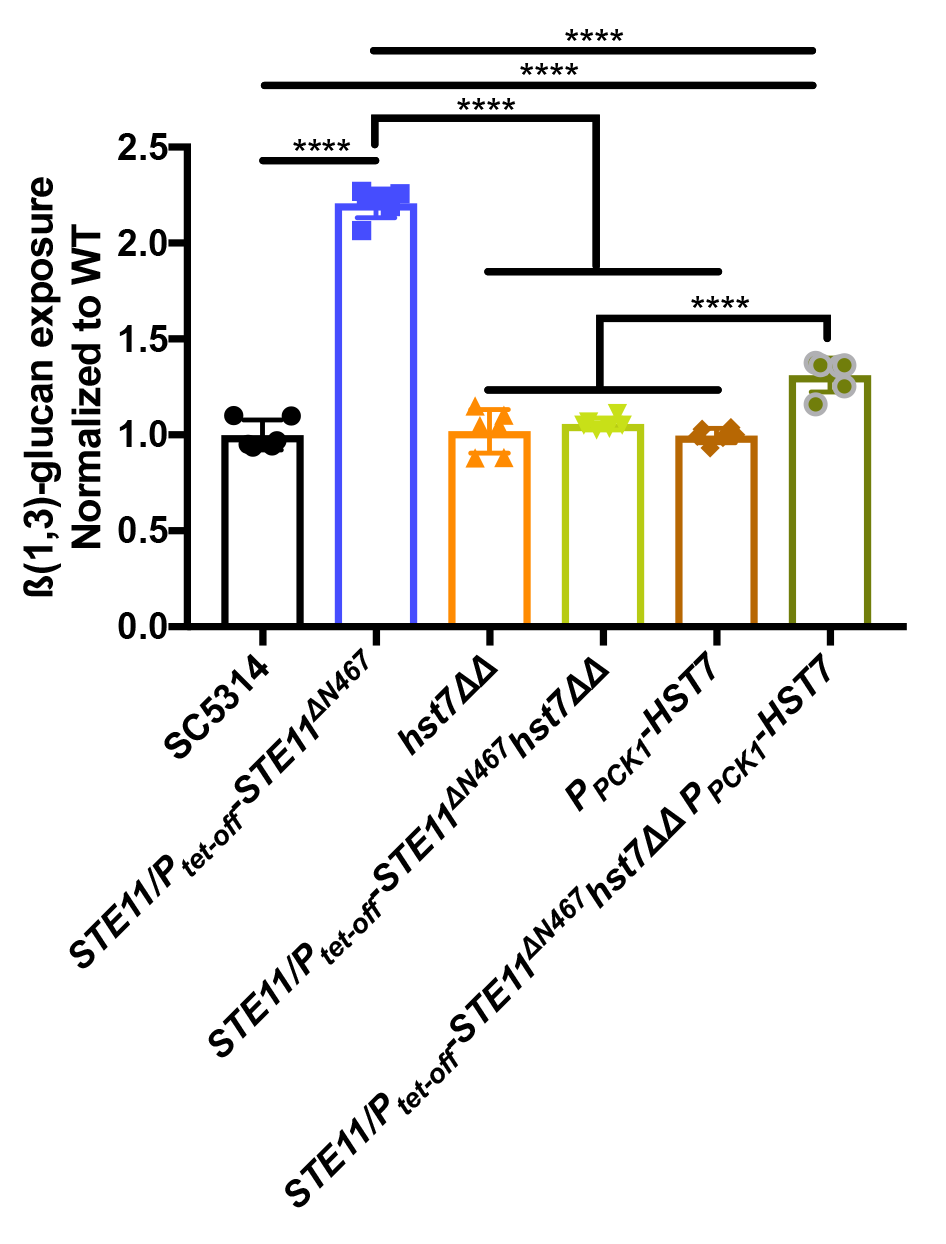

Supplement: S7 Fig — Overnight cultures of the wild-type and hst7ΔΔ mutants were stained with an anti-ß(1,3)-glucan antibody and a phycoerythrin-conjugated secondary antibody followed by flow cytometry analysis to assess the levels of ß(1,3)-glucan exposure (****p<0.0001, by one-way ANOVA with Tukey’s post hoc analysis). (TIF) [file ppat.1009839.s007.tif]
