# Supplementary material for: Activation of Cph1 causes ß(1,3)-glucan unmasking in Candida albicans and attenuates virulence in mice in a neutrophil-dependent manner
Source: PLoS Pathog. 2021 Aug 25;17(8):e1009839. doi: 10.1371/journal.ppat.1009839 (PMC8423308; doi:10.1371/journal.ppat.1009839)
Supplement: S1 Table — (DOCX) [file ppat.1009839.s008.docx]

**S1 Table: Plasmids used in this study.**

| **Strain Name** | **Description** | **Markers (Bacterial, Fungal)** | **Parent/Source** |
| --- | --- | --- | --- |
| pYLC146 | *FLP-CaNAT* | Chloramphenicol, Nourseothricin | [1] |
| pBT1 | *CaNAT-P_ENO1_* | Ampicillin, Nourseothricin | [2] |
| pADH110 | CRISPR fragment A template | Ampicillin, Nourseothricin* | [3] |
| pADH119 | CRISPR fragment B template | Ampicillin, Nourseothricin* | [3] |
| pADH137 | CRISPR Cas9 expression Plasmid | Ampicillin, Nourseothricin* | [3] |
| pAW006 | *LEU2 5'UTR-FLP-CaNAT* | Chloramphenicol, Nourseothricin | pYLC146 |
| pAW011 | *FLP-CaNAT-LEU2* Knockout Plasmid | Chloramphenicol, Nourseothricin | pAW006 |
| pTC074 | FLP-CaNAT-*CPH1* 3'UTR | Chloramphenicol, Nourseothricin | pYLC146 |
| pAW054 | *CaNAT-Peno1-CPH1* | Ampicillin, Nourseothricin | pBT1 |
| pAW066 | *CaNAT-Peno1-DFI1* | Ampicillin, Nourseothricin | pBT1 |
| pAW071 | *FLP-CaNAT*-*CPH1-flag* reintegrate plasmid | Chloramphenicol, Nourseothricin | pTC074 |
| pAW085 | *FLP-CaNAT*-*PCK1* 3'UTR | Chloramphenicol, Nourseothricin | pYLC146 |
| pAW087 | *FLP-CaNAT*-*P_PCK1_* overexpression cassette | Chloramphenicol, Nourseothricin | pAW085 |
| pAW091 | *FLP-CaNAT*-*P_PCK1-_CEK1* | Chloramphenicol, Nourseothricin | pAW087 |
| pAW105 | *FLP-CaNAT-P _PCK1_-HST7* | Chloramphenicol, Nourseothricin | pAW087 |

*These plasmids each function together to make a split nourseothricin gene that comes together to form a functional open reading frame after successful transformation and recombination into the *Candida albicans LEU2* locus.

**References:**

1. Chen YL, Montedonico AE, Kauffman S, Dunlap JR, Menn FM, Reynolds TB. Phosphatidylserine synthase and phosphatidylserine decarboxylase are essential for cell wall integrity and virulence in Candida albicans. Mol Microbiol. 2010;75(5):1112-32. Epub 2010/02/06. doi: 10.1111/j.1365-2958.2009.07018.x. PubMed PMID: 20132453.

2. Tams RN, Cassilly CD, Anaokar S, Brewer WT, Dinsmore JT, Chen YL, et al. Overproduction of Phospholipids by the Kennedy Pathway Leads to Hypervirulence in Candida albicans. Front Microbiol. 2019;10:86. Epub 2019/02/23. doi: 10.3389/fmicb.2019.00086. PubMed PMID: 30792701; PubMed Central PMCID: PMCPMC6374345.

3. Nguyen N, Quail MMF, Hernday AD. An Efficient, Rapid, and Recyclable System for CRISPR-Mediated Genome Editing in Candida albicans. mSphere. 2017;2(2). Epub 2017/05/13. doi: 10.1128/mSphereDirect.00149-17. PubMed PMID: 28497115; PubMed Central PMCID: PMCPMC5422035.
