# Supplementary material for: Activation of Cph1 causes ß(1,3)-glucan unmasking in Candida albicans and attenuates virulence in mice in a neutrophil-dependent manner
Source: PLoS Pathog. 2021 Aug 25;17(8):e1009839. doi: 10.1371/journal.ppat.1009839 (PMC8423308; doi:10.1371/journal.ppat.1009839)
Supplement: S3 Table — (DOCX) [file ppat.1009839.s010.docx]

**S3 Table: Strains used in this study.**

| **Strain** | **Genotype** | **Parent** | **Source or Reference** |
| --- | --- | --- | --- |
| SC5314 | Prototrophic Wild Type | Clinical Isolate | [1] |
| DAY286 | ura3::imm434/ura3::imm434, his1::hisG/his1::hisG, pARG4::URA3::arg4::hisG/arg4::hisG) | SC5314 | [2] |
| CAI-4 | *ura3*::imm434/*ura3*::imm434 *iro1/iro1*::imm434 | SC5314 | [3] |
| TC147 | *STE11/P_tet-off_-STE11^∆N467^* | Day286 | [4] |
| AWY006 | *LEU2/leu2∆* (Wild Type) | SC5314 | This Study |
| AWY080 | *STE11/P_tet-off_-STE11^∆N467^* *LEU2/leu2∆* | AWY006 | This Study |
| AWY109 | *P_ENO1_-CPH1 LEU2/leu2∆* | AWY006 | This Study |
| AWY112 | *P_ENO1_-DFI1 LEU2/leu2∆* | AWY006 | This Study |
| AWY144 | *STE11/P_tet-off_-STE11^∆N467^cph1∆∆ LEU2/leu2∆* | AWY080 | This Study |
| AWY154 | *STE11/P_tet-off_-STE11^∆N467^cph1∆∆::CPH1-Flag LEU2/leu2∆* | AWY144 | This Study |
| AWY173 | *opy2∆∆ LEU2/leu2∆* | AWY006 | This Study |
| AWY174 | *STE11/P_tet-off_-STE11^∆N467^*opy2∆∆ LEU2/leu2∆ | AWY080 | This Study |
| AWY182 | *cph1∆∆ LEU2/leu2∆* | AWY006 | This Study |
| AWY183 | *dfi1∆∆ LEU2/leu2∆* | AWY006 | This Study |
| AWY184 | *opy2∆∆ dfi1∆∆ LEU2/leu2∆* | AWY173 | This Study |
| AWY187 | *STE11/P_tet-off_-STE11^∆N467^* *dfi1∆∆ LEU2/leu2∆* | AWY080 | This Study |
| AWY188 | *STE11/P_tet-off_-STE11^∆N467^* *opy2∆∆ dfi1∆∆ LEU2/leu2∆* | AWY174 | This Study |
| AWY208 | *cek1∆∆ LEU2/leu2∆* | AWY006 | This Study |
| AWY212 | *STE11/P_tet-off_-STE11^∆N467^* *dfi1∆∆ P_ENO1_-DFI1 LEU2/leu2∆* | AWY187 | This Study |
| AWY215 | *STE11/P_tet-off_-STE11^∆N467^* *opy2∆∆ dfi1∆∆ P_ENO1_-DFI1 LEU2/leu2∆* | AWY188 | This Study |
| AWY220 | *STE11/P_tet-off_-STE11^∆N467^* *P_ENO1_-DFI1 LEU2/leu2∆* | AWY080 | This Study |
| AWY236 | *STE11/P_tet-off_-STE11^∆N467^* *cek1∆∆ LEU2/leu2∆* | AWY080 | This Study |
| AWY221 | *cek1∆∆ leu2∆∆* | CAI-4 | This Study |
| AWY240 | *P_PCK1_-CEK1 LEU2/leu2∆* | AWY006 | This Study |
| AWY242 | *STE11/P_tet-off_-STE11^∆N467^* *cek1∆∆ P_PCK1_-CEK1 LEU2/leu2∆* | AWY236 | This Study |
| AWY264 | *hst7∆∆ LEU2/leu2∆* | AWY006 | This Study |
| AWY266 | *STE11/P_tet-off_-STE11∆N467 hst7∆∆ LEU2/leu2∆* | AWY080 | This Study |
| AWY270 | *STE11/P_tet-off_-STE11^∆N467^* *hst7∆∆ P_PCK1_-HST7 LEU2/leu2∆* | AWY266 | This Study |
| AWY272 | *P_PCK1_-HST7 LEU2/leu2∆* | AWY264 | This Study |

**References:**

1. Gillum AM, Tsay EY, Kirsch DR. Isolation of the Candida albicans gene for orotidine-5'-phosphate decarboxylase by complementation of S. cerevisiae ura3 and E. coli pyrF mutations. Mol Gen Genet. 1984;198(2):179-82. Epub 1984/01/01. doi: 10.1007/BF00328721. PubMed PMID: 6394964.

2. Davis DA, Bruno VM, Loza L, Filler SG, Mitchell AP. Candida albicans Mds3p, a conserved regulator of pH responses and virulence identified through insertional mutagenesis. Genetics. 2002;162(4):1573-81. Epub 2003/01/14. PubMed PMID: 12524333; PubMed Central PMCID: PMCPMC1462392.

3. Fonzi WA, Irwin MY. Isogenic strain construction and gene mapping in Candida albicans. Genetics. 1993;134(3):717-28. Epub 1993/07/01. PubMed PMID: 8349105; PubMed Central PMCID: PMCPMC1205510.

4. Chen T, Wagner AS, Tams RN, Eyer JE, Kauffman SJ, Gann ER, et al. Lrg1 Regulates beta (1,3)-Glucan Masking in Candida albicans through the Cek1 MAP Kinase Pathway. mBio. 2019;10(5). Epub 2019/09/19. doi: 10.1128/mBio.01767-19. PubMed PMID: 31530671; PubMed Central PMCID: PMCPMC6751057.
